# Assessing uranium and select trace elements associated with breccia pipe uranium deposits in the Colorado River and main tributaries in Grand Canyon, USA

**DOI:** 10.1371/journal.pone.0241502

**Published:** 2020-11-04

**Authors:** Fred D. Tillman, Jessica R. Anderson, Joel A. Unema, Thomas P. Chapin

**Affiliations:** 1 U.S. Geological Survey, Arizona Water Science Center, Tucson, Arizona, United States of America; 2 U.S. Geological Survey, Arizona Water Science Center, Flagstaff, Arizona, United States of America; 3 U.S. Geological Survey, Geology, Geophysics and Geochemistry Science Center, Denver, Colorado, United States of America; Chinese Academy of Sciences, CHINA

## Abstract

Assessing chemical loading from streams in remote, difficult-to-access watersheds is challenging. The Grand Canyon area in northern Arizona, an international tourist destination and sacred place for many Native Americans, is characterized by broad plateaus divided by canyons as much as two-thousand meters deep and hosts some of the highest-grade uranium deposits in the U.S. From 2015–2018 major surface waters in Grand Canyon were monitored for select elements associated with breccia-pipe uranium deposits in the area, including uranium, arsenic, cadmium, and lead. Dissolved constituents in the Colorado River were monitored upstream (Lees Ferry), in the middle (Phantom Ranch), and downstream (Diamond Creek) of uranium mining areas. Concentrations of uranium, arsenic, cadmium, and lead at these main-stem sites varied little during the study period and were all well below human health and aquatic life benchmark criteria (30, 10, 5, and 15 μg/L maximum contaminant levels and 15, 150, 0.8, and 3.1 μg/L aquatic life criteria, respectively). Additionally, dissolved and sediment-bound constituents were monitored during a wide range of streamflow conditions at Little Colorado River, Kanab Creek, and Havasu Creek tributaries, whose watersheds have experienced different levels of uranium mining activities over time. Samples from the tributary sites contained ≤3.8 μg/L of dissolved cadmium and lead, and ≤17 μg/L of dissolved uranium. Dissolved arsenic also was mostly below human and aquatic life criteria at Little Colorado River and Kanab Creek; however, 63% of water samples from Havasu Creek were above the maximum contaminant level for arsenic. Arsenic in suspended sediment was greater than sediment quality guidelines in 9%, 35%, and 35% of samples from Little Colorado River, Kanab Creek, and Havasu Creek, respectively. At the concentrations observed during this study, tributaries contributed on average only about 0.12 μg/L of arsenic and 0.03 μg/L of uranium to the main-stem river. This study demonstrates how chemical loading from mined watersheds may be reliably assessed across a wide range of flow conditions in challenging locations.

## Introduction

Uranium provinces are found in locations throughout the world [1]. In the decade 2004–2014, the largest conventional uranium deposits were discovered in the countries of Namibia, Niger, Mongolia, Canada, and Australia [2]. If uranium mining facilities are poorly planned or operated, environmental effects may occur when natural events such as seasonal runoff or intense rainfall lead to off-site movement of metals. Compiling comprehensive baseline environmental data prior to beginning uranium mining operations is critical to determining if environmental effects occur after operations have begun [3]. In some locations, uranium mines operate in remote areas with sometimes difficult monitoring conditions. In the United States (U.S.), over 4,000 uranium mining locations with documented production are located in the mostly rural western states of Colorado, New Mexico, Utah, Wyoming, and Arizona, typically on Federal and Tribal lands [4]. The Grand Canyon area in northern Arizona is one such active uranium mining region.

**Fig 1:**
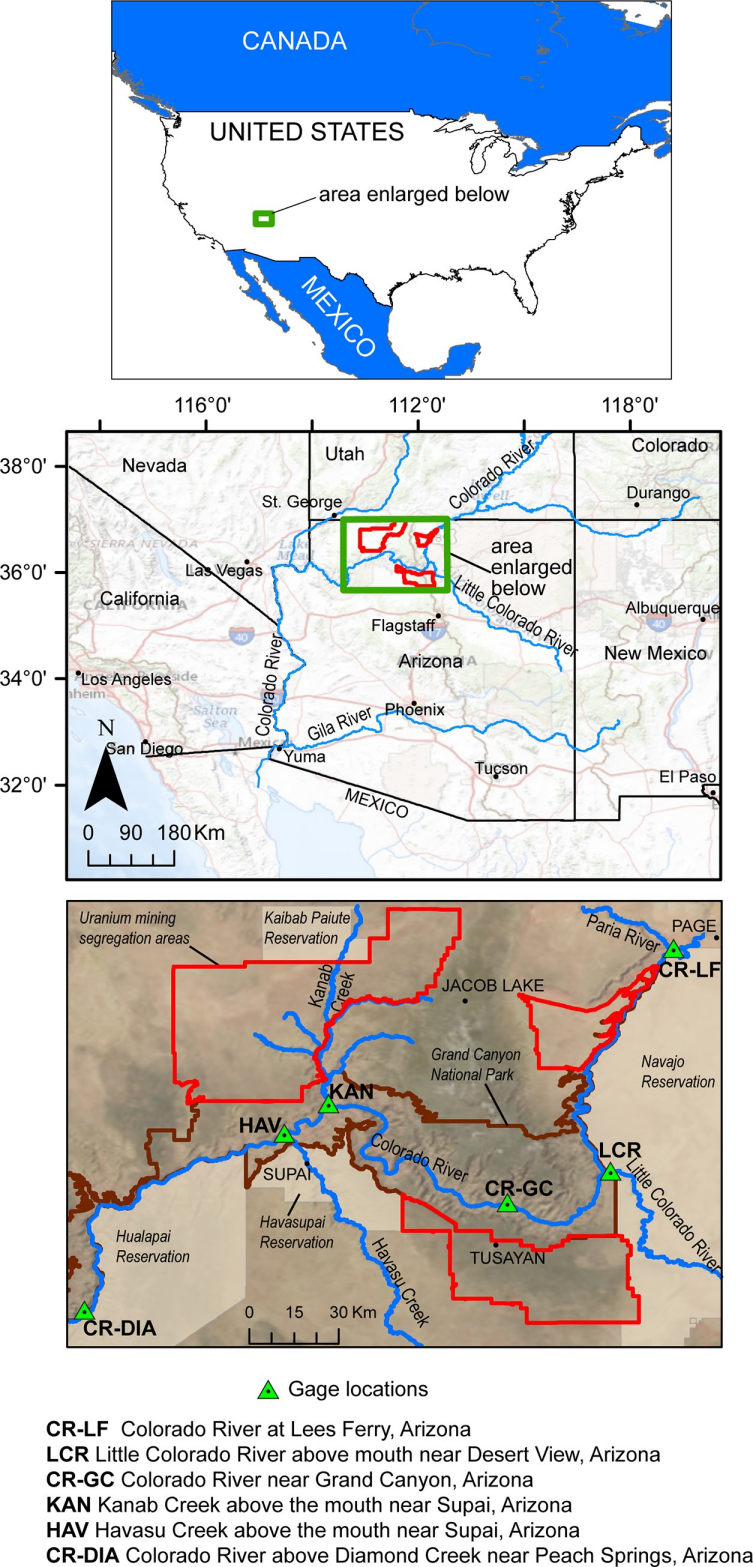
Regional (upper panel) and local (lower panel) view of study area. Map data from the USGS including the USGS National Map.

Grand Canyon in Arizona is a United Nations World Heritage Site [5] and a tourist destination for over six million people each year. The Grand Canyon region is a home or sacred place of origin for many Native Americans and its cultural significance goes back thousands of years. The Colorado River, which carved Grand Canyon, is a primary source of drinking and irrigation water for 35 million people in the U.S. and Mexico [6]. The Grand Canyon region also hosts some of the highest-grade uranium ore in the United States [7]. In 2012, then-U.S. Secretary of the Interior Ken Salazar signed a Record of Decision (ROD) to withdraw over 1 million acres in three segregation areas of Federal land in the Grand Canyon region from new uranium mining activities for the next 20 years, subject to valid existing rights (Fig 1) [8]. A key factor in the decision for the withdrawal was the limited amount of scientific data and resulting uncertainty on potential effects of uranium mining activities on cultural, biological, and water resources in the area. Since 2014, the U.S. Geological Survey (USGS) has planned and conducted scientific investigations to address the uncertainties of potential uranium-mining effects noted in the ROD. Investigations related to regional water resources include understanding what constitutes “background” or naturally occurring concentrations of uranium and associated trace elements in groundwater [9–11], developing a conceptual model of the regional and perched groundwater systems in the area, and investigating the concentration of uranium and associated trace elements in the Colorado River and major tributaries (this manuscript).

Uranium mining in the Grand Canyon area dates to the early 1950s when uranium ore was found in the old workings at the Orphan copper mine on the South Rim of Grand Canyon [12]. Uranium ore in this area is mined from collapse features called breccia pipes, which are chimney-like geologic formations filled with rubble (breccia). There are thousands of collapse features in the Grand Canyon area, many of which are breccia pipes, and some, but not all, of these are mineralized with uranium ore [13]. Along with uranium, other associated trace elements may be enriched in mineralized breccia pipes including silver, arsenic, barium, cadmium, cobalt, copper, mercury, molybdenum, nickel, lead, antimony, strontium, vanadium, and zinc [14]. Many of these elements also occur naturally in rock units and surface soils in the area, but at substantially lower concentrations [15]. While no effects on water resources from modern mining practices in the area have been demonstrated conclusively to date, exposing ore bodies and waste rock from breccia pipes to oxidized water could potentially result in transport of some metals away from mine sites through groundwater, with subsequent discharge to springs and rivers. Additionally, metals in dust around mine sites and haul roads may dissolve or be entrained in runoff, ultimately moving down drainages to tributaries and the Colorado River [16].

This paper presents results from an investigation of the concentration of uranium and associated trace elements in the main stem of the Colorado River upstream, in the middle, and downstream of Grand Canyon National Park and major breccia-pipe mining areas, as well as in three major tributaries (Little Colorado River, Kanab Creek, and Havasu Creek). For the period 2015–2018, concentrations of uranium, arsenic, cadmium, and lead were monitored in water and suspended sediment at the tributary sites under baseflow and runoff conditions, and for dissolved-only concentrations at the main-stem Colorado River monitoring sites. The concentration data collected for this study are combined with streamflow and suspended-sediment discharge data to estimate the load of uranium, arsenic, cadmium, and lead entering the Colorado River from the main tributaries in Grand Canyon during select periods encompassing both baseflow and runoff conditions. Understanding the concentrations of uranium and associated trace elements in water at these locations is important to establish baseline conditions against which to compare future monitoring data should widespread uranium mining return to the area. With continued monitoring at these and other sites in the area, important comparisons between mined and unmined drainages, or between before and after mining activities, can be made to better understand the potential for effects on water resources from uranium mining in the area. Additionally, methods and analyses used in this investigation may be helpful in evaluating potential water-quality changes at other remote, difficult-to-monitor uranium mining locations.

### Previous studies

While several studies have been published on water quality of groundwater discharging from springs in the Grand Canyon region [e.g., 9, 10, 17–19], few published studies have focused on the concentration of metals in the Colorado River and major tributaries that are related to uranium mining in the area. Hopkins et al. [20] collected water and stream sediment samples in March of 1982 in the Snake Gulch drainage of Kanab Creek as part of determining mineral resource potential in the area. No samples were collected at the mouth of Kanab Creek. Foust and Hoppe [21] presented original and previously published results from individual water-quality sampling events over a ten-year period of waters in Grand Canyon National Park, including samples from Havasu Creek, Kanab Creek, and the Little Colorado River. A suite of trace element results was presented in the report, but the list does not include uranium. Stewart et al. [22] presented radionuclide concentrations (uranium isotopes and radium) from Grand Canyon water samples including one sample each in July and September of 1986 at the Little Colorado River at the mouth, a July 1986 sample at Kanab Creek, and August 1985 and July 1986 samples at Havasu Creek. Fisk et al. [23] presented results from a four-year study of water and sediment quality in the Little Colorado River basin, with a focus on radionuclides that might be related to uranium mining and milling operations in the basin. Samples were collected from several sites within the Little Colorado River basin, the most downstream being at Little Colorado River near Cameron, Arizona, which is some 70 river kilometers upstream from the Colorado River. In 1996, the U.S. National Park Service published a compilation of water quality data from sites in and near Grand Canyon National Park that were available from U.S. Environmental Protection Agency databases [24]. Data were collected by State and Federal agencies, including USGS, and included samples from Lees Ferry, Little Colorado River, Kanab Creek, and Havasu Creek, among others. Limited uranium analyses were included for the Colorado River and major tributaries including no uranium data for Lees Ferry, three results for Little Colorado River, two samples from Kanab Creek, and two samples from Havasu Creek. Taylor et al. [25] detailed two water-quality studies conducted by USGS on the Colorado River in Grand Canyon. Water samples were collected at 10 main-stem and 6 tributary sites every 6 hours for a 48-hour period in November 1990 and June 1991. Results from Taylor et al. [25] are discussed further in the “Results and discussion” section of this paper. None of the aforementioned studies provided extended water and suspended sediment quality data over both baseflow and runoff conditions, nor estimated load of uranium and associated trace elements in streams and rivers in the area.

## Methods and materials

### Study area and monitoring sites

**Fig 2:**
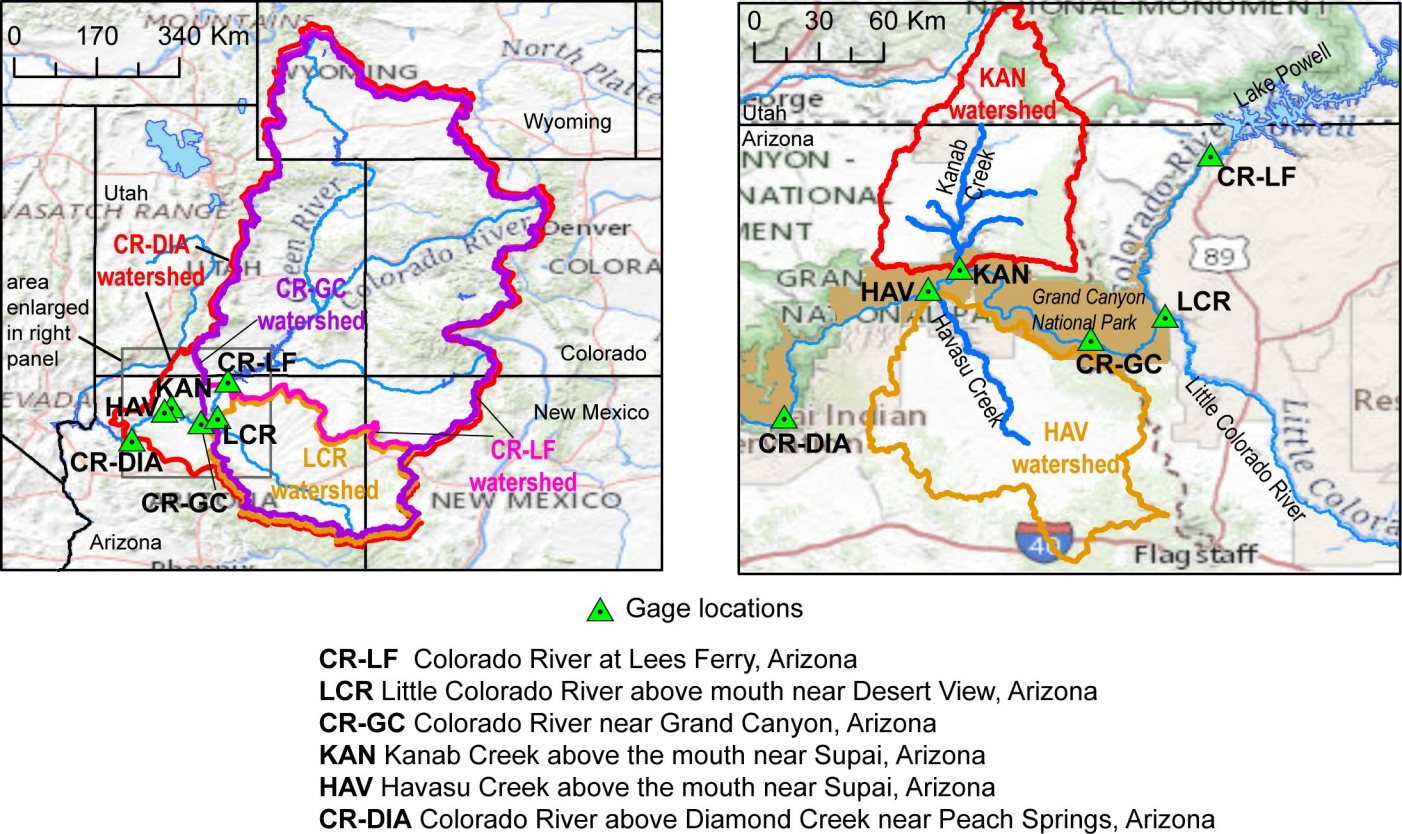
Watershed boundaries of three main-stem Colorado River monitoring sites (left panel) and three tributary monitoring sites (left and right panels) discussed in this study. Note that watersheds for all three main-stem Colorado River sites include the upper Colorado River basin and therefore overlap in the left panel. Map data from the USGS including the USGS National Map.

The concentration of dissolved uranium and select associated trace elements was monitored at three sites on the main stem of the Colorado River (Fig 2, Table 1). Colorado River at Lees Ferry, Arizona (USGS site ID 09380000; hereafter Lees Ferry) and Colorado River above Diamond Creek near Peach Springs, Arizona (USGS site ID 09404200; hereafter Colorado River–Diamond Creek) represent upstream and downstream conditions of watersheds with breccia pipe uranium mining locations in the area, with Colorado River near Grand Canyon, Arizona (USGS site ID 09402500; hereafter Colorado River–Grand Canyon) monitoring concentrations in the river near the midpoint of the study area. Lees Ferry and Colorado River–Grand Canyon were monitored with permission from the U.S. National Park Service (Research Permit GRCA-2019-SCI-0007) and Colorado River–Diamond Creek was monitored with permission from the Hualapai Tribe (Administrative Permit 0011). The Lees Ferry monitoring site (S1 Fig in S1 File) is 25 km downstream from Glen Canyon Dam and provides water-quality information for the Colorado River before the river flows through breccia pipe uranium mining areas. Since 1963, streamflow at Lees Ferry has been regulated by Glen Canyon Dam and releases from Lake Powell, and averages about 380 m^3^/s [26]. The USGS National Water Quality Assessment program (NAWQA) monitors water quality at the Lees Ferry site about 12 times per year. The Colorado River–Grand Canyon monitoring site (S2 and S3 Figs in S1 File), located near Phantom Ranch in Grand Canyon National Park, is 155 km downstream from the Lees Ferry monitoring site. Streamflow at the Colorado River–Grand Canyon site averages about 390 m^3^/s [26], integrating Colorado River flow with flow from the Little Colorado and Paria River tributaries, and ephemeral flow from side canyons such as Rider Canyon. Dissolved constituents are monitored at Colorado River–Grand Canyon once per year. The Colorado River–Diamond Creek site (S4 Fig in S1 File) is 364 km downstream from the Lees Ferry monitoring site and downstream of all uranium mining segregation areas. Streamflow at the Colorado River–Diamond Creek site integrates inputs from all other sites in this study and is the last monitoring site on the Colorado River before flow enters Lake Mead. Streamflow at this site averages about 395 m^3^/s [26]. The USGS monitors water quality at the Colorado River–Diamond Creek site about twice per year with support from the Arizona Department of Environmental Quality.

**Table 1:** Details of monitoring sites in Grand Canyon used for this study.

USGS site number	Station name	Drainage area (km^2^)	Gage elevation (m NGVD29^1^)	Remarks
09380000	COLORADO RIVER AT LEES FERRY, AZ	289,561	939	25 km downstream from Glen Canyon dam. Gage monitors integrated flow from entire upper Colorado River basin, minus diversions.
09402300	LITTLE COLORADO RIVER ABV MOUTH NR DESERT VIEW, AZ	69,857	841	Monitors areas with dispersed uranium mining and includes flow from Puerco River, site of uranium tailings spill^2^.
09402500	COLORADO RIVER NEAR GRAND CANYON, AZ	366,742	737	Monitors upper Colorado River basin, Lower Colorado River, and Paria River drainage areas.
09403850	KANAB CREEK ABOVE THE MOUTH NEAR SUPAI, AZ	6,131	585	Kanab Creek drainage area includes six current and former breccia pipe uranium mining complexes^3^.
09404115	HAVASU CREEK ABOVE THE MOUTH, NEAR SUPAI, AZ	7,822	549	Flow almost entirely from discharge of Havasu Springs. Drainage area includes Canyon Mine uranium mine.
09404200	COLORADO RVR ABV DIAMOND CREEK NR PEACH SPRINGS AZ	386,727	408	Integrates flow from above 5 gages plus additional drainage along the Colorado River.

^1^ meters above the National Geodetic Vertical Datum of 1929

^2^ Widespread mining of roll front uranium deposits on Navajo Land [27]; spill of uranium mine tailings pond from Church Rock uranium mill in 1979 [28].

^3^ Former mines include Kanab North Mine, Hermit Mine, Pigeon Mine, and Hack Mine complex; current mines include Pinenut Mine (in reclamation) and Arizona 1.

Uranium and associated trace element concentrations in both water and suspended sediment were monitored at three major tributary inputs to the Colorado River between the upstream and downstream main-stem sites (Fig 2, Table 1): Little Colorado River above the mouth near Desert View, Arizona (USGS site ID 09402300); Kanab Creek above the mouth near Supai, Arizona (USGS site ID 09403850); and Havasu Creek above the mouth near Supai, Arizona (USGS site ID 09404115). Little Colorado River, Kanab Creek, and Havasu Creek were monitored with permission from the U.S. National Park Service (Research Permit GRCA-2019-SCI-0007). Streamflow at all three tributary monitoring sites in Grand Canyon is characterized by perennial baseflow with periods of intense, high suspended-sediment runoff of limited duration. The Little Colorado River site (S5 Fig in S1 File) drains the Navajo Nation Reservation that has experienced widespread, disseminated uranium mining in the past that is unrelated to breccia-pipe uranium mining [27]. The Little Colorado River also receives flow from the Puerco River, along which the Church Rock uranium mill spill occurred in 1979 [28]. The Little Colorado River enters the Colorado River about 100 km downstream of the Lees Ferry monitoring site and about 45 km upstream of the Colorado River–Grand Canyon monitoring site (Fig 1). Streamflow at the Little Colorado River site averages about 6 m^3^/s, although runoff events periodically produce short-term flow as high as 100 m^3^/s [26]. Kanab Creek enters the Colorado River about 102 km downstream of the Colorado River–Grand Canyon monitoring site (Fig 1). The Kanab Creek site (S6 and S7 Figs in S1 File) monitors flow from a drainage area containing planned, currently active, and reclaimed breccia pipe uranium mines [13]. Streamflow at the Kanab Creek monitoring site averages about 0.25 m^3^/s, with heavy suspended-sediment runoff events producing limited-term flows as high as 150 m^3^/s [26]. Havasu Creek enters the Colorado River about 127 km downstream of the Colorado River–Grand Canyon monitoring site and about 25 km downstream from the mouth of Kanab Creek (Fig 1). The Havasu Creek monitoring site (S8 and S9 Figs in S1 File) is at the mouth of Havasu Creek where it enters the Colorado River, about 7 km downstream from Supai Village. Havasu Creek flows through Cataract Canyon in the heart of the Havasupai Reservation, is fed by Havasu Springs, and is world-famous for its blue-green waterfalls (S10 Fig in S1 File). The drainage area for the Havasu Creek site contains both active (Canyon Mine) and planned (Wate Mine) breccia pipe uranium mines. Streamflow at the Havasu Creek monitoring site averages about 2 m^3^/s with storm-generated flow over 100 m^3^/s recorded in July 2018 [26].

### Monitoring data

Water samples were collected on the main stem of the Colorado River upstream, near the midpoint, and downstream of Grand Canyon National Park at the Lees Ferry, Colorado River–Grand Canyon, and Colorado River–Diamond Creek monitoring sites, respectively. Water samples at these main-stem sites were collected using the equal discharge increment method (EDI). EDI is an isokinetic, depth-integrating method that ensures that each unit of stream discharge is equally represented in the combined sample. Samples were collected in HDPE (high-density polyethylene) bottles, filtered immediately after collection using a 0.45 μm capsule filter, and preserved to pH<2 with Ultrex grade nitric acid. All sampling equipment was cleaned following USGS protocols [29] for inorganic constituents including 30 minute soak and scrub in 2% liquinox detergent, 3+ rinses with tap water, 30 minute soak in 5% HCl solution, and 3+ rinses with deionized water. Filtered and preserved water samples were shipped to the USGS National Water Quality Laboratory in Denver, Colorado, for metals and trace element analyses.

Water and suspended sediment samples were collected at three tributary sites in Grand Canyon: Little Colorado River, Kanab Creek, and Havasu Creek, using programmable ISCO autosamplers and by grab sampling. Suspended sediment was collected and analyzed in addition to water at these sites because of the high suspended-sediment loads coming from the tributaries [30] and the tendency of radionuclides and other constituents to sorb to fine-grained sediment [23]. The remoteness of the tributary sites allows only limited access (normally twice per year), mostly by boat trips on the Colorado River or by helicopter. Grab samples were collected and processed onsite when field personnel visited the monitoring sites and autosamplers were used to collect samples during the remaining times, particularly during higher flow events.

Autosamplers at these sites were programmed to collect combined water and suspended-sediment samples during flood stages into 1 L polypropylene bottles that were cleaned following USGS protocols [29] for sampling inorganic constituents (30 minute soak and scrub in 2% liquinox detergent, 3+ rinses with tap water, 30 minute soak in 5% HCl solution, and 3+ rinses with deionized water). Autosamplers were triggered by data collection platforms when the river stage rose to a threshold value 0.3 to 0.46 m (1 to 1.5 ft) (variable by site) above baseflow. Additional samples were then triggered while flow remained elevated above the threshold every 2–48 hours (variable by site) and when stage values rose or fell 0.15–0.61 m (0.5–2.0 ft) (variable by site). Grab samples were collected directly from the stream and processed (syringe filtration with 0.45-μm Luer-Lok filter, preservation with ACS grade nitric acid) immediately at the monitoring sites at the time of autosampler bottle retrieval and new bottle deployment to monitor concentrations during typically lower flow conditions. Autosampler bottles containing water and suspended sediment were capped at the time of sample retrieval and processed at the USGS facility in Flagstaff, Arizona. First, suspended sediment was allowed to settle out of the water, then a 0.45-μm filtered water sample was collected from each bottle by syringe and preserved with ACS grade nitric acid. Water samples from the tributary sites were shipped to the USGS Geology, Geophysics, and Geochemistry Science Center in Denver, Colorado, for analyses. Next, most of the remaining water in the autosampler bottles was carefully decanted, avoiding any visible discharge of suspended sediment. The remaining suspended sediment slurry was poured into pre-cleaned 600-mL polypropylene beakers and allowed to air dry. The dried sediment was shipped to USGS contract laboratory AGAT Laboratories in Ontario, Canada, for elemental analyses.

Filtered main-stem water samples were analyzed for major ions and trace elements by the USGS National Water Quality Laboratory using inductively coupled plasma–mass spectrometry (ICP-MS) and ion chromatography (IC) [31, 32]. Tributary water samples were analyzed by the USGS Geology, Geophysics, and Geochemistry Science Center using ICP-MS [33]. Suspended sediment analyses were conducted by AGAT Laboratories for 49 major, minor, and trace elements [34]. Suspended sediment samples were first decomposed using a mixture of hydrochloric, nitric, perchloric, and hydrofluoric acids at low temperature. The resulting solution was analyzed by ICP optical emission spectrometry (OES) and ICP-MS. Suspended sediment concentrations from environmental samples are compared with sediment quality guidelines (Table 2) that were developed for bioavailable (weak-acid digestion) concentrations. The total digestion method used in this study results in a maximum estimate of metals associated with suspended sediment and may overestimate available metal concentrations. Comparing total digestion concentrations with weak-acid digestion guidelines represents a conservative approach for this investigation. The analytical methods used for water and suspended sediment elemental analyses produce results for a suite of elements. This investigation focuses on results for select elements that are potentially associated with breccia pipe uranium deposits in the region, have relatively low benchmark values (i.e., primary drinking water standard values or aquatic life criteria values; Table 2), and are available from ICP-MS analyses. These elements include uranium, arsenic, cadmium, and lead and results are provided in the Supplemental Materials. Full analytical results for all water and suspended-sediment samples are available from USGS ScienceBase [35].

**Table 2:** Drinking water-quality standards, aquatic toxicity reference values, and sediment quality guidelines for elements investigated in this study.

Element	Primary drinking-water standard (MCL^1^) [μg/L]	Aquatic life criteria^2^ [μg/L]		Sediment quality guidelines^5^ [mg/kg]
		Acute exposure	Chronic exposure	
Arsenic	10	340	150	9.79
Cadmium	5	2.2–5.2^3^	0.8–1.7^3^	0.99
Lead	15	80.2–218.9^3^	3.1–8.5^3^	35.8
Uranium	30	33^4^	15^4^	104.4

^1^Maximum contaminant level; U.S. Environmental Protection Agency [36].

^2^U.S. Environmental Protection Agency [37], except uranium (Canadian Council of Ministers of the Environment [38]).

^3^Criteria are dependent on the hardness of the water at the site. Range presented are lowest (Kanab Creek) and highest (Little Colorado River) values for the study area.

^4^Unfiltered water samples.

^5^Consensus-based threshold effect concentration (MacDonald et al. [39]) except uranium, which is the lowest effect level using the weighted method in Thompson et al. [40].

The load of uranium and select trace elements was estimated at the tributary monitoring sites during selected time periods with both baseflow and runoff conditions using concentration data collected for this study. The estimation of load (mass per time) requires information on element concentration (mass per volume) and discharge (volume per time). Loading of dissolved and suspended-sediment-associated elements was estimated at all three tributary monitoring sites, requiring filtered water sample concentration, concentration of metals in suspended sediment, streamflow, and sediment discharge data. The USGS Grand Canyon Monitoring and Research Center (GCMRC) and the USGS Arizona Water Science Center maintain streamflow and suspended-sediment load monitoring stations at several locations in the Grand Canyon region, including Little Colorado River, Kanab Creek, and Havasu Creek. Streamflow data for all sites in this study were obtained from the USGS National Water Information System [26]. Sediment discharge data at the three tributary monitoring sites in this study were obtained from the USGS GCMRC database [30].

The pH and redox conditions at the monitoring sites, in addition to the presence of complexing ions, largely controls expected mobility of arsenic, cadmium, lead, and uranium in water and suspended sediment [41]. Monitoring data from main-stem and tributary sites [26] indicate neutral to slightly alkaline (7.9–8.4) pH and oxic redox conditions. Arsenic in water forms the stable oxyanions arsenate (As^5+^) and arsenite (As^3+^). At the pH conditions of the monitoring sites, the divalent arsenate anion HAsO_4_^2-^ is expected to predominate [42]. Because of its negative charge, the arsenate anion is more strongly sorbed to mineral surfaces than arsenite, resulting in less mobility in water [43], although several commonly occurring anions in natural waters may compete with arsenic for mineral sorption sites [41]. Adsorption by hydrous iron oxide can limit concentrations of arsenic to low levels in water [44]. In pH 8 oxic waters, dissolved cadmium exists as both the uncomplexed Cd^2+^ ion and the carbonate complex CdCO_3_ [45, 46]. While cadmium readily adsorbs to suspended particulate matter, high concentrations of competing divalent cations (i.e., Ca^2+^, Mg^2+^) and complexation with carbonates and chlorides may reduce the transfer of cadmium from the aqueous phase to suspended sediments [47]. For lead, PbCO_3_ is the dominant aqueous species in oxic pH 8 waters, but dissolved lead concentrations tend to be very low due to the limited solubility of lead salts (carbonates, sulfates, phosphates) [45]. Dissolved lead also has a very high affinity for sorption to particulate matter and rarely desorbs under oxic pH 8 conditions. Therefore, transfer of lead from sediments to the aqueous phase is retarded [46]. At the neutral-to-slightly alkaline pH and oxic conditions encountered at the monitoring sites, uranium, as the uranyl ion, UO_2_^2+^, readily forms highly mobile carbonate complexes [48].

### Quality control

While water quality samples at the three main-stem Colorado River monitoring sites were collected and processed immediately according to USGS protocols [29], water samples collected by autosampler at the three tributary sites in Grand Canyon require interpretation of associated quality control (QC) samples to be properly evaluated. Ensuring the quality of water samples from the autosampler-monitored tributary sites is challenging owing to the extreme range in streamflow from baseflow to flood flows and the remoteness of the monitoring sites, leading to potentially long time periods between sample collection and sample retrieval and processing. Bottle filling in the autosamplers during high flood flow may be accompanied by splashing that can cause transfer of mass between bottles. Water samples collected early during a deployment period may sit in uncapped sample bottles inside the autosampler for weeks to months before being retrieved, resulting in evaporation and concentration of elemental mass in remaining water. As solute concentrations increase, however, the evaporation rate of the sample will be reduced relative to that of pure water in accordance with Raoult’s Law [49]. Mixtures of suspended sediment and water inside sample bottles may undergo partitioning that result in their concentrations being different at time of sample processing than what would be observed at time of sample collection. As described above, arsenic in the form of the arsenate anion, may sorb to mineral surfaces [43] and dissolved lead has a high affinity for sorption to particulate matter [46].

To evaluate the potential effects of these and other processes on samples collected by autosampler, new QC methods and reference samples were developed and employed. QC processes evolved and developed over time, so not all QC results are available for all autosampler deployment periods. At the time of deployment, autosamplers were prepared with one uncapped 1-L bottle containing inorganic blank water and one uncapped 1-L bottle containing a metals reference standard of known concentrations. Blank sample analyses provide an indication of cross-contamination within the autosampler and during sample transportation, and the reference standard analyses indicate how evaporation may have affected water sample concentrations during deployment. Additionally, during deployment a grab sample was collected and processed from the stream while the autosampler was triggered. A sample was immediately collected by syringe from the autosampler bottle that was triggered. In this way, the similarity between an autosampler-collected stream sample and a grab stream sample can be evaluated. The triggered bottle was left uncapped in the autosampler for the duration of the deployment and subsequently collected and processed with the rest of the environmental samples collected during the deployment. By comparing results to the initial sample concentration in the autosampler bottle at the time of deployment, the final concentration in the bottle is a check on combined evaporation and potential partitioning effects on concentrations of natural samples (i.e., stream water mixed with suspended sediment) in autosampler bottles.

A laboratory evaluation of the USGS Geology, Geophysics, and Geochemistry Science Center laboratory was performed to understand variability in analytical results and how this variability might affect interpretation of tributary dissolved concentrations. Over a period of two years, five natural-water samples of known metal concentrations were sent for analyses to the USGS Geology, Geophysics, and Geochemistry Science Center lab as part of the USGS Branch of Quality Systems “round robin” quality assurance program. Finally, two laboratory experiments were conducted to better understand the potential for partitioning between suspended sediment and water in autosampler bottles. In one experiment, dried sediment was re-suspended in deionized water for 3–4 weeks, with before and after suspended sediment and water samples collected and compared. A second experiment collected time-series water samples over a two-month period from a bottle containing a natural water sample from the Little Colorado River monitoring site (see Supporting Information for methodology and results).

### Statistical methods

Descriptive, correlative, and hypothesis-testing statistics were performed using the R statistical platform [50]. The NADA package [51] was used for datasets with censored (i.e., less-than-reporting limit) values. To compute descriptive statistics and boxplots for datasets with censored values, the imputation method “regression on order statistics” (ROS) was used. The ROS method fills in non-detect data based on a probability plot of values above detection limits and can incorporate datasets with multiple reporting limits [52, 53]. Tests of correlation were performed using Kendall’s tau, which is a nonparametric test that can be applied to datasets with multiple censored levels [53]. To compare results between the three tributary monitoring sites, the Peto-Prentice version of the generalized nonparametric Wilcoxon test was used for datasets with censored results [53] and the Kruskal-Wallis analysis of ranks was used for datasets with results all above reporting limits [54]. A significant statistical test result was defined as having a p-value <0.05.

## Results and discussion

### Quality control for tributary water samples

Evaluation of analytical variability of the USGS Geology, Geophysics, and Geochemistry Science Center laboratory resulted in mean percent differences between laboratory-reported and known concentrations of 14% or less for the four focus elements for the five natural water laboratory evaluation samples (S1 Table in S1 File). Of the 20 elemental comparisons, only 2 were reported with differences above 25%. Cadmium from one sample had a percent difference of 39% (0.62 μg/L reported versus 0.42 μg/L actual) and a lead sample had a percent difference of 28% (0.25 μg/L reported versus 0.33 μg/L actual). Reported results from tributary sites for these elements, therefore, should be considered uncertain in the low sub-μg/L range.

Results from available autosampler blanks were generally below reporting limits for all focus elements, with a few exceptions (S2–S4 Tables in S1 File, S11–S13 Figs in S1 File). Cadmium was detected in three blanks, all at concentrations of ≤0.7 μg/L (S2 and S3 Tables in S1 File). Lead was detected in four autosampler blanks, all at concentrations of ≤0.9 μg/L. Finally, uranium was detected at a concentration of 0.6 μg/L in a single blank from the Little Colorado River site. If detectable concentrations in an autosampler blank were observed during a deployment, then the environmental samples collected during that deployment were evaluated and rejected if they were less than 4 times the concentration in the blank.

Elemental results from stream grab samples and autosampler triggered samples were comparable for nearly all of the 19 co-collected samples. All co-collected stream grab samples and autosampler triggered samples were within 1 μg/L of each other for all elements (97%), with the exception of one arsenic (9.9 and 11.8 μg/L, respectively) and one lead (<0.2 and 3.8 μg/L, respectively) pair from the Havasu Creek site (S2–S4 Tables in S1 File, S11–S13 Figs in S1 File). It is likely that contamination contributed to the high lead result from the autosampler as the 3.8 μg/L value is over 3 times the next highest lead value observed at the site and over 17 times the mean lead concentration. Additionally, all other elements analyzed during the same stream and autosampler comparison were within 0.9 μg/L of each other (S4 Table in S1 File). For the most part, autosampler collected samples were equivalent to stream grab samples, providing confidence in the sample collection process.

Reference samples prepared in deionized water that were left uncapped in the autosamplers for the duration of their deployment were nearly all higher in concentration for all focus elements at the end of the deployment than they were at the beginning, as would be expected from sample evaporation (S2–S4 Tables in S1 File, S11–S13 Figs in S1 File). Of the 56 before-and-after-deployment elemental comparisons of reference samples, only 6 did not indicate a possible concentrating effect from evaporation. For all 6 of these comparisons, the before and after deployment results were within 4.5% (percentage change–difference divided by initial value) or less of each other, within analytical variability determined by the laboratory evaluation program, suggesting that the true evaporative effect may have been negligible in these samples. To provide an estimate of the potential effects of evaporation on environmental samples collected in autosamplers, a potential percent increase in concentration is estimated based on the number of days between the day of sample collection and the day of sample retrieval using the average rate of change in concentrations for all focus elements during the autosampler deployment (S8 Table in S1 File). If deployment-specific evaporation rates from reference samples were not available for a deployment with environmental samples, then average rates computed from reference samples deployed during a similar time of year (season) were used. The potential effects of evaporation on sample results are discussed separately for each tributary site in the following sections.

Autosamplers were triggered at the time of deployment, with a syringe sub-sample collected from the triggered autosampler bottle for comparison with a stream grab sample, as described previously. The bottle containing the triggered sample remained uncapped in the autosampler until the following sample retrieval trip. By comparing end- and beginning-of-deployment concentrations in a natural water sample, both evaporation and partitioning processes can be evaluated. Of the 65 elemental comparisons from before-and-after natural water samples, 11 indicated lower concentrations at the end of deployment than at the beginning, contrary to most results from the evaporation QC samples (S2–S4 Tables in S1 File, S11–S13 Figs in S1 File). Of these decreasing-concentration samples, 3 were for arsenic (maximum decrease of 2.6 μg/L), 2 were for cadmium (maximum decrease of 0.7 μg/L), 1 was for lead (maximum decrease of 0.4 μg/L), and 5 were for uranium (maximum decrease of 3.6 μg/L). Partitioning from dissolved to solid phases is expected for cadmium and lead, and possibly arsenic, in the pH and redox conditions at the tributary sites, but uranium partitioning is unexpected. Because aqueous phase concentration decreases are not observed routinely during autosampler deployments, it is difficult to establish a quantitative estimate for potential effects on environmental sample concentrations as was done for the evaporation QC samples. Results from laboratory experiments conducted to evaluate the potential for partitioning in autosampler bottles indicated small amounts of arsenic, lead, and uranium may enter deionized water solution from suspended sediment (S5 and S6 Tables in S1 File) and an irregular decrease in arsenic concentration in a natural water sample over time (S7 Table in S1 File). Taken together, these results suggest that there is the potential for exchange or transformation of elemental mass in autosampler bottles, but the magnitude is most likely limited to single μg/L concentrations. By the time samples are collected at tributary monitoring sites, stream water and suspended sediment have had extended contact time and have likely reached an equilibrium in elemental mass between aqueous and solid phases; thus, further transfer between phases in open sample bottles is probably limited. Implications of partitioning in autosampler bottles on confidence in environmental sample concentrations are discussed separately for each tributary site.

### Main-stem Colorado River monitoring results

**Fig 3:**
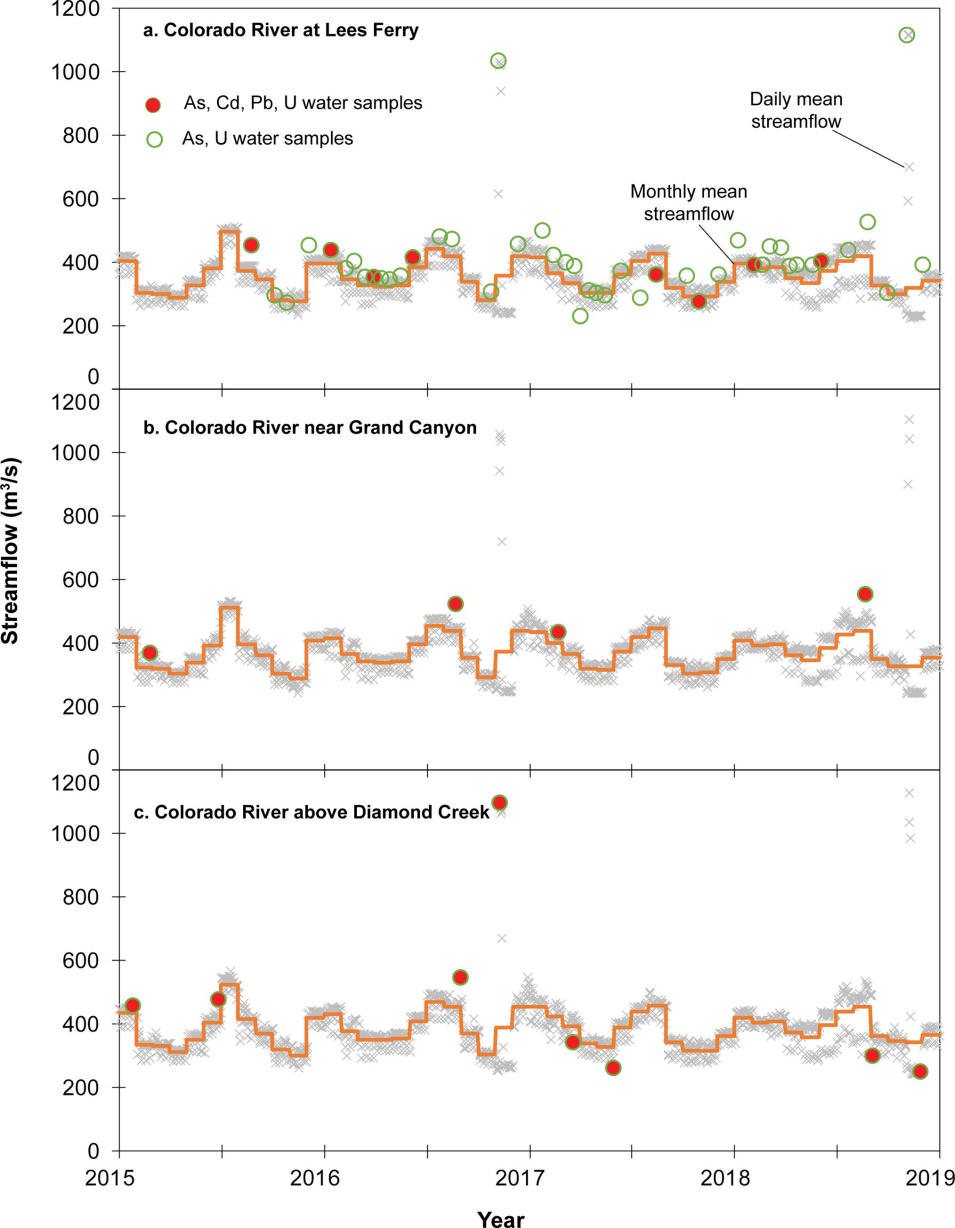
Daily and monthly average streamflow with water quality sample times for three main-stem Colorado River monitoring sites. Samples are plotted with streamflow at time of sample collection, which may be greater or lesser than daily mean streamflow value.

**Fig 4:**
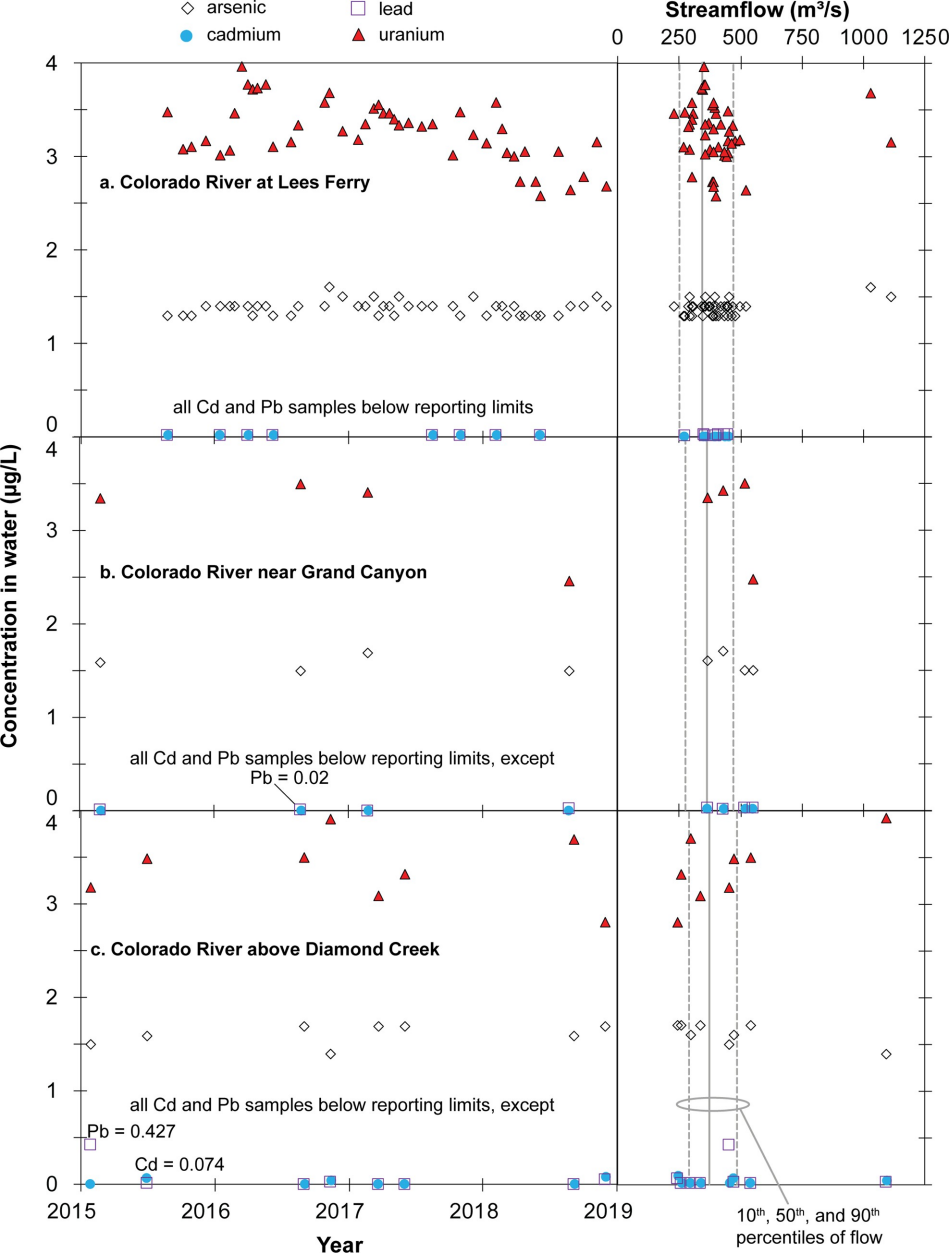
Observed concentrations of arsenic, cadmium, lead, and uranium in water samples from main-stem Colorado River monitoring sites in Grand Canyon during the 2015–2018 study period. Censored results plotted at half the reporting limit.

During the 2015–2018 time period of this investigation, 46 filtered water samples were collected at the Lees Ferry monitoring site (Table 3; Fig 3A), 4 filtered water samples were collected at the Colorado River–Grand Canyon site (Fig 3B), and 8 filtered water samples were collected at the Colorado River–Diamond Creek site (Fig 3C). Of the 46 water samples collected at Lees Ferry, all were analyzed for both arsenic and uranium, with 8 of these samples also analyzed for cadmium and lead. Streamflow on the Colorado River below Lake Powell is almost entirely controlled by releases from Glen Canyon Dam and, with the notable exception of High-Flow Experimental (HFE) releases in November 2016 and November 2018, varies within a limited range throughout the study period (Fig 3A). Water samples were collected at Lees Ferry across the limited range of flow conditions experienced at the site, from less than the 10^th^ percentile of flow to more than the 90^th^ percentile of flow and included samples during both of the HFE releases (Figs 3A and 4A). All water samples from Lees Ferry contained detectable concentrations of arsenic and uranium, but no sample results for cadmium or lead were above reporting limits (Fig 4A; S8 Table in S1 File). Arsenic concentrations ranged from 1.3 μg/L to 1.6 μg/L with a mean of 1.4 μg/L, all well below the maximum contaminant level (MCL) of 10 μg/L and the aquatic life chronic exposure criteria of 150 μg/L (Table 2). Uranium concentrations were somewhat higher than arsenic concentrations, ranging from 2.6 to 3.9 μg/L with a mean value of 3.3 μg/L (Fig 4A). All uranium results were well below the MCL of 30 μg/L and below the aquatic life chronic exposure criteria of 15 μg/L (Table 2). Neither arsenic nor uranium concentrations appear to vary substantially through time (Fig 4A left panel) or with streamflow (Fig 4A right panel), including concentrations from samples collected during the HFE releases.

**Table 3:** Number of water and suspended sediment samples collected at each monitoring site during the 2015–2018 time period of this study, in downstream order.

Monitoring site	Site type	Number of water samples	Number of suspended-sediment samples
Colorado River at Lees Ferry	main stem	46^1^	NS^2^
Little Colorado River above the mouth	tributary	103	106
Colorado River–Grand Canyon	main stem	4	NS
Kanab Creek above the mouth	tributary	57	23
Havasu Creek above the mouth	tributary	38	17
Colorado River–Diamond Creek	main stem	8	NS

^1^46 water samples collected for As and U analyses, 8 of these also analyzed for Cd and Pb.

^2^NS: not sampled. Suspended sediment samples not collected on main stem Colorado River sites.

Fewer samples were collected at the Colorado River–Grand Canyon monitoring site, owing to the difficulty of accessing and sampling the site (S2 and S3 Figs in S1 File). Four water samples were collected at the site during the study period, mostly during median or higher streamflow conditions (Figs 3B and 4B). Arsenic values ranged from 1.5 to 1.7 μg/L with a mean of 1.5 μg/L and uranium values ranged from 2.4 to 3.5 μg/L with a mean of 3.1 μg/L, all below benchmark values (Table 2). Low cadmium and lead concentrations were observed, with only a single sample containing lead equal to the reporting limit (0.02 μg/L; Fig 4B). It is difficult to observe trends in time or with streamflow at the site, based on so few sample results (Fig 4B). Concentrations of all focus elements are similar between the Lees Ferry and Grand Canyon monitoring sites, indicating little sustained effects from tributary inputs to the main stem in this reach.

At the Colorado River–Diamond Creek monitoring site, 8 water samples were collected during the study period, at a range of streamflow conditions from less than the 10^th^ percentile of flow to more than the 90^th^ percentile of flow and included a sample during the HFE release in 2016 (Figs 3C and 4C). As with other main-stem sites, cadmium and lead were mostly below reporting limits, with the exception of a single sample of lead of 0.4 μg/L and another sample with cadmium of 0.07 μg/L (Fig 4C). Arsenic values ranged from 1.4 to 1.7 μg/L with a mean of 1.6 μg/L. Uranium in water samples from the site ranged from 2.8 to 3.9 μg/L with a mean of 3.3 μg/L. Results for all samples were well below drinking water and aquatic life standards (Table 2). Similar to observed concentrations at Lees Ferry, there is no apparent trend in concentrations with either time or streamflow at the Colorado River–Diamond Creek monitoring site (Fig 4).

### Tributary monitoring results

**Fig 5:**
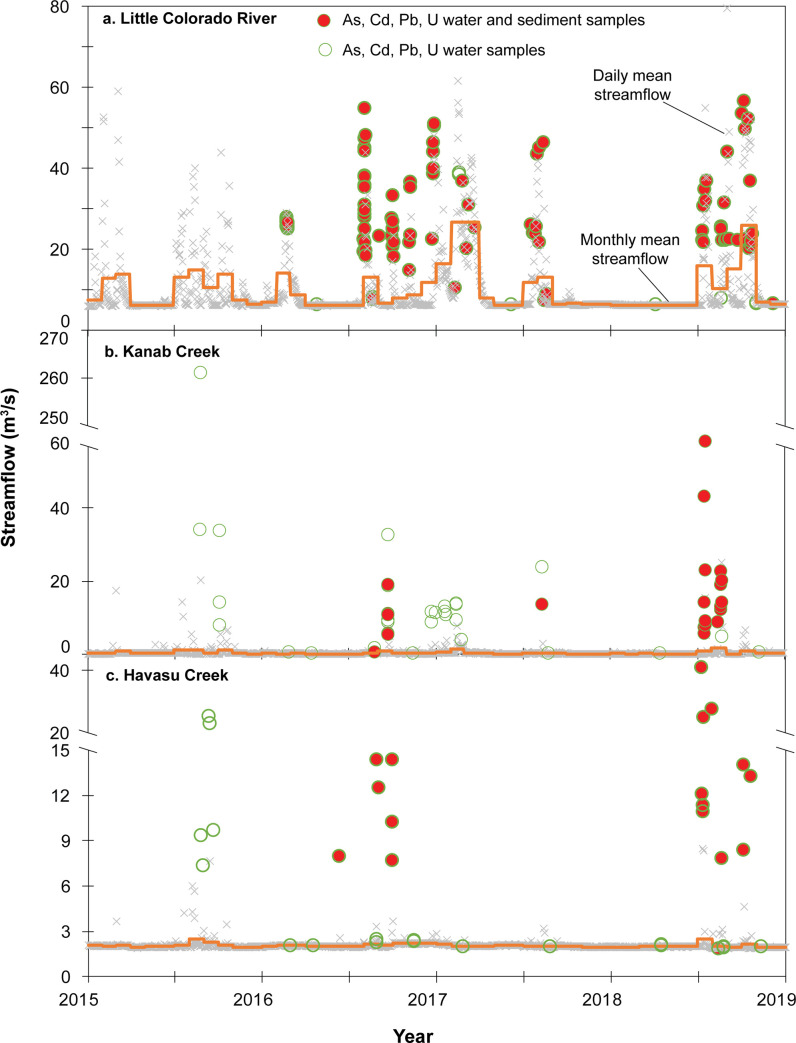
Daily and monthly average streamflow with water quality and suspended sediment sample times for three tributary monitoring sites. Samples are plotted with streamflow at time of sample collection, which may be greater or lesser than daily mean streamflow value.

**Fig 6:**
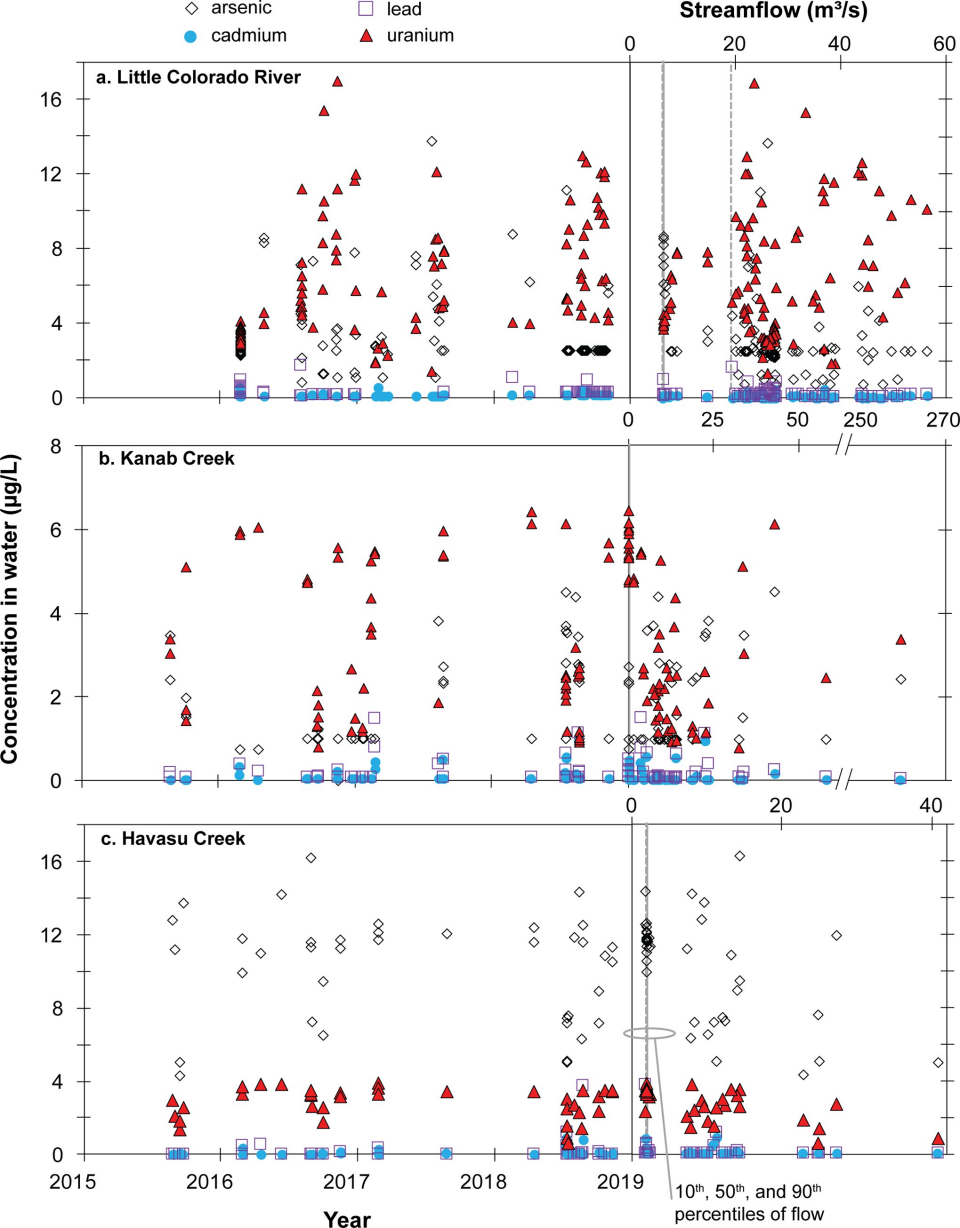
Observed concentrations of arsenic, cadmium, lead, and uranium in water samples from tributary monitoring sites in Grand Canyon during the 2015–2018 study period. Censored results plotted at half the reporting limit.

**Fig 7:**
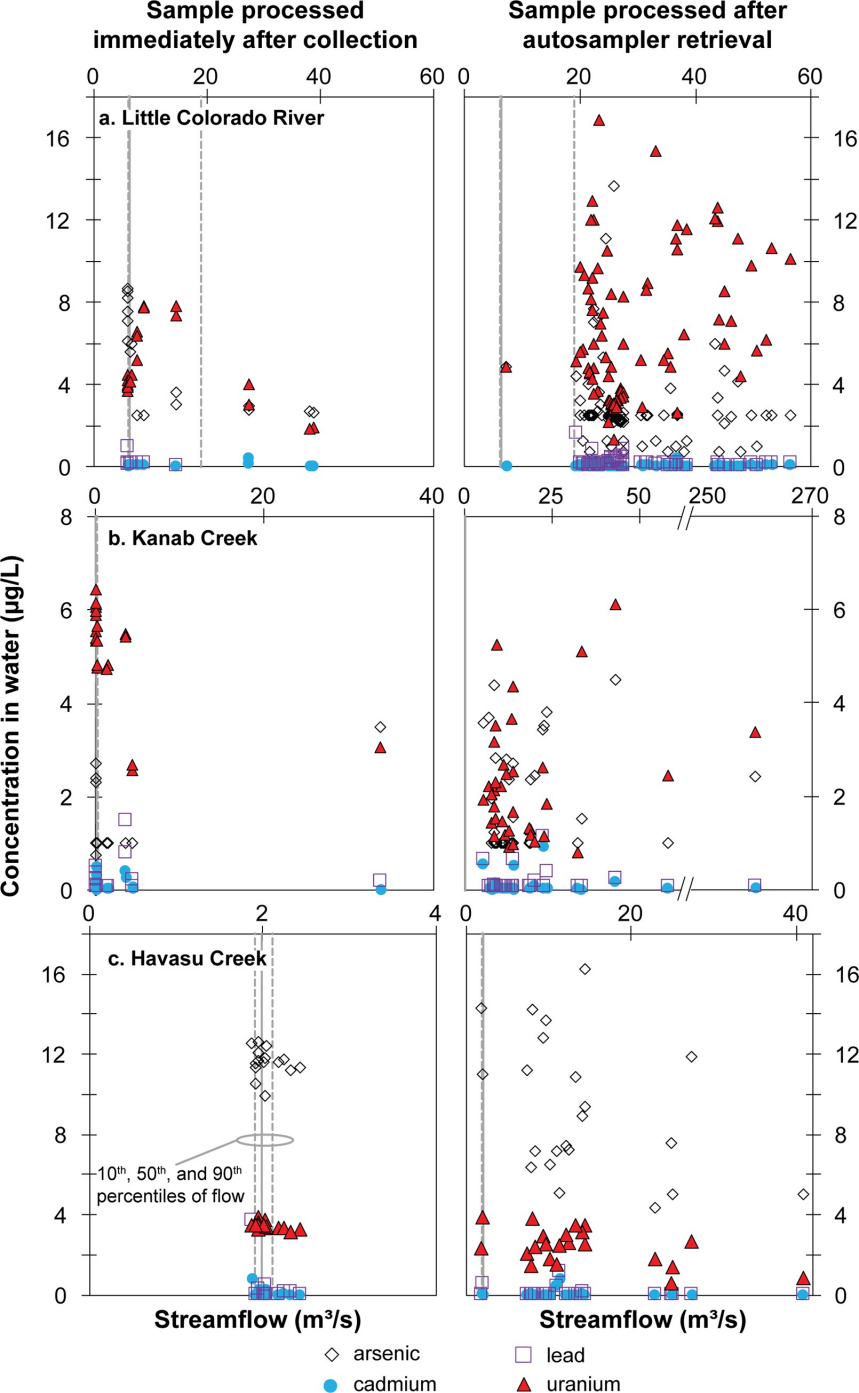
Comparison of arsenic, cadmium, lead, and uranium concentrations in water samples from tributary monitoring sites in Grand Canyon between samples processed immediately after collection and samples processed after autosampler retrieval. Censored results plotted at half the reporting limit.

**Fig 8:**
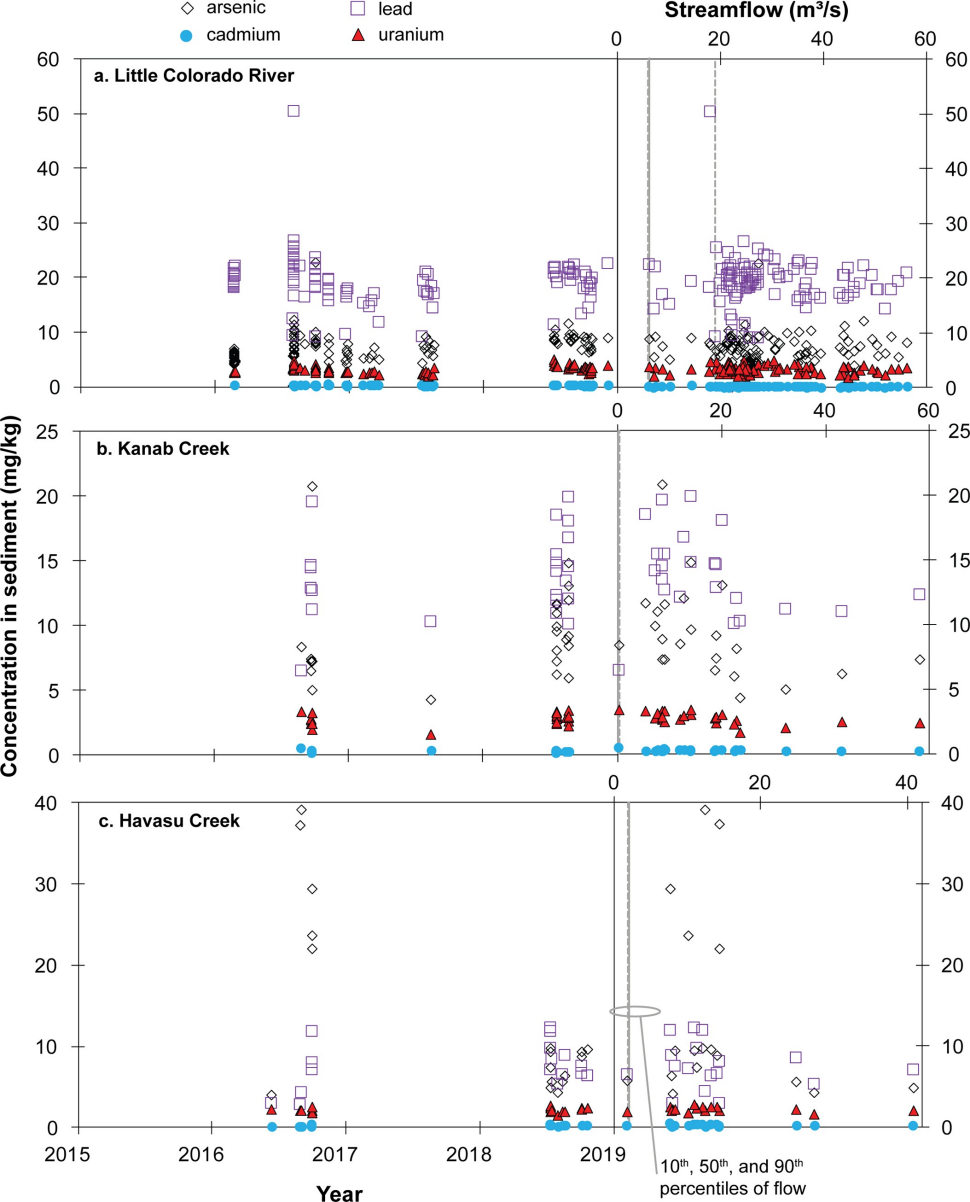
Observed concentrations of arsenic, cadmium, lead, and uranium in suspended sediment samples collected with water at tributary monitoring sites in Grand Canyon during the 2015–2018 study period.

**Fig 9:**
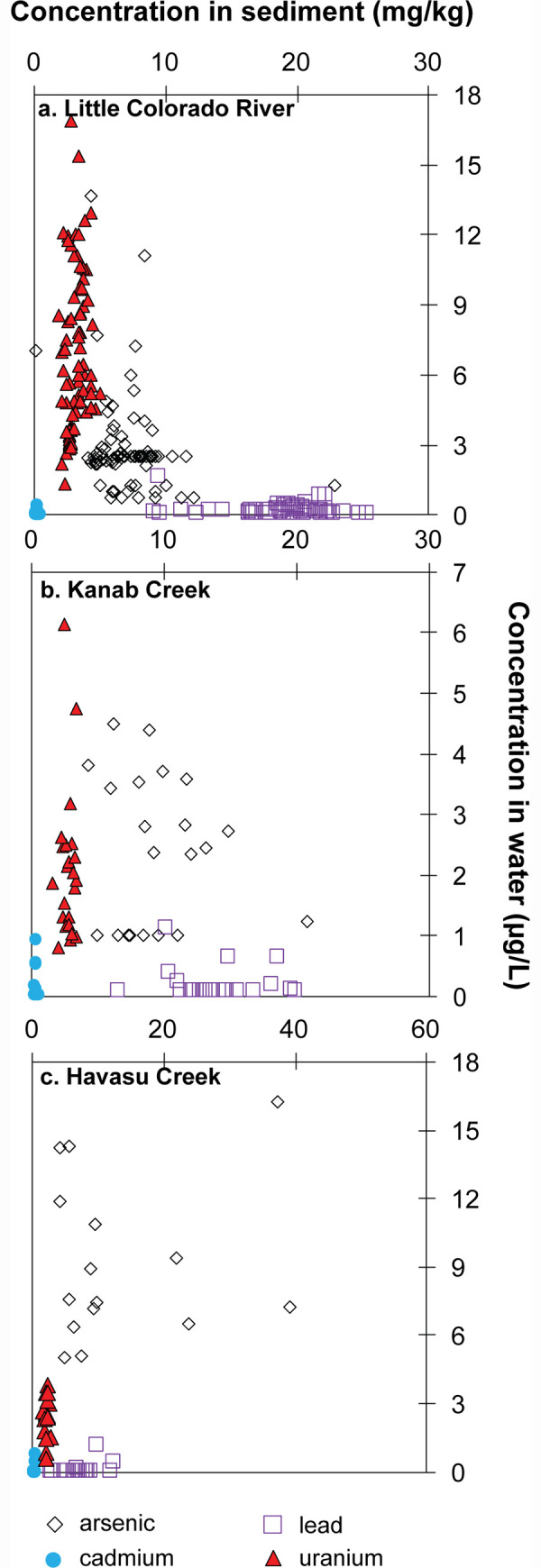
Comparison of water and suspended sediment concentrations of arsenic, cadmium, lead, and uranium from co-collected samples at tributary monitoring sites in Grand Canyon. Censored results plotted at half the reporting limit.

Both water and suspended sediment samples were collected at the Little Colorado River, Kanab Creek, and Havasu Creek tributary sites in Grand Canyon (Figs 5–9). Results from each tributary site are discussed separately.

#### Little Colorado River monitoring site

During the 2015–2018 time period of this investigation, 103 water samples and 106 suspended-sediment samples were collected at the Little Colorado River monitoring site during a range of flow conditions from baseflow to over 5 times daily mean flow (Table 3; Fig 5A). As with the main-stem water results, cadmium and lead concentrations were largely below reporting limits, with 97% of cadmium samples and 73% of lead samples with less-than-reporting-limit results (S8 Table in S1 File; Fig 6A). The highest reported cadmium concentration at the Little Colorado River site was 0.5 μg/L and the highest measured lead concentration was 1.7 μg/L, both substantially below MCLs of 5 μg/L and 15 μg/L, respectively, and below chronic exposure aquatic life criteria of 1.7 μg/L and 8.5 μg/L, respectively (Table 2). Observed concentrations of cadmium and lead in the Little Colorado River were comparable to mean values at the site reported by Taylor et al. [25] of <0.02 μg/L and 0.38 μg/L, respectively. Limited dissolved cadmium and lead are expected at the neutral to slightly alkaline pH and oxic conditions at the Little Colorado River monitoring site [46, 47]. Detectable arsenic concentrations were reported in most water samples (57%) and detectable uranium concentrations were reported in all 103 water samples (S8 Table in S1 File). The median concentration of arsenic in water samples was 2.5 μg/L and there were 2 water samples with concentrations greater than the 10 μg/L MCL, with a highest concentration of 13.7 μg/L (S8 Table in S1 File; Fig 6A). Uranium concentrations in water in the Little Colorado River ranged from 1.3 μg/L to 17 μg/L, with a median of 5.2 μg/L during the study period. No samples were above the uranium MCL of 30 μg/L, and only 2 samples had uranium concentrations greater than the chronic exposure aquatic life criteria of 15 μg/L (Table 2). Median concentrations of arsenic and uranium in Little Colorado River water samples were less than reported mean values of 6.7 μg/L and 9.2 μg/L, respectively, in Taylor et al. [25].

As discussed in the QC methods section, evaporation and possible partitioning within autosampler collected samples may alter concentrations measured at the time of sample analysis from the concentration in the stream at the time of sample collection. Based on QC sample results, potential increase in environmental sample concentrations from evaporation was estimated to be as high as 14%, based on before-and-after deployment QC samples and time before environmental sample retrieval, for samples collected at the Little Colorado River monitoring site (S8 Table in S1 File). Taken together or individually, increasing length of time between sample collection and retrieval is not significantly correlated with increasing sample concentrations (S14 Fig in S1 File). Overall, evaporation from autosampler bottles would have the effect of increasing measured sample concentrations over the concentration in the stream at the time of sample collection. Arsenic and uranium mass loss from water in QC autosampler bottles was observed during 4 out of 24 pre-and-post deployment comparisons (twice each), with a maximum decline of 3 μg/L. Concentrations in environmental water samples collected by autosampler during these three deployments may underestimate risk relative to benchmarks. There is, however, no evidence of substantial environmental sample concentration declines in results for arsenic and uranium. Environmental samples collected during deployments when one or more QC samples indicated a decline in autosampler concentrations had fewer samples with less-than-reporting-limit concentrations for arsenic (33% versus 53% when no QC sample concentration declined) and had higher mean concentrations for both arsenic (3.6 μg/L versus 2.7 μg/L) and uranium (7.3 μg/L versus 6.2 μg/L). The combined effects of evaporation and limited partitioning from water may serve to moderate each other for some elements. Of the 106 water samples collected at the Little Colorado River site, 19 of them (18%) were processed onsite and thus not subject to evaporation or partitioning in autosampler bottles (Fig 7). Median concentrations of all focus elements were comparable between onsite and autosampler samples (3.7 versus 2.5 μg/L for arsenic, 0.03 versus 0.002 μg/L for cadmium, 0.18 versus 0.19 μg/L for lead, and 4.2 versus 5.6 μg/L for uranium). Samples processed onsite at the Little Colorado River indicate declining concentrations of uranium with higher flows (Fig 7), so higher uranium concentrations in some autosampler-collected samples may be a result of the effects of evaporation. However, other relatively high uranium values may be true representations of in-stream concentrations. For example, 4 of the highest 12 observed uranium concentrations were collected by autosampler in November and December of 2016 during which time little evaporation is expected owing to cooler temperatures. In fact, no change in uranium concentration was measured in the reference QC sample that remained in the autosampler for the entire deployment (S2 Table in S1 File).

Suspended-sediment results from 106 samples from the Little Colorado River monitoring site were above reporting limits for all elements, with the exception of a single arsenic sample in August of 2016 (S8 Table in S1 File; Fig 8A). In contrast with water samples from the site, suspended-sediment concentrations were highest for lead with a median concentration of 19.5 mg/kg (S8 Table in S1 File; Fig 8A). Dissolved lead has a high affinity for sorption to particulate matter and rarely desorbs under oxic pH 8 conditions found at the site [46]. However, only a single lead sample (50.3 mg/kg) was above the sediment quality guideline of 35.8 mg/kg (Table 2). Suspended-sediment concentrations of lead in the Little Colorado River were mostly greater than the median value of 13 mg/kg for surface soils of the Colorado Plateau in the Grand Canyon region reported by Van Gosen [15], but no sample had a lead concentration greater than the 170-mg/kg maximum value in Van Gosen [15]. Arsenic concentrations in suspended sediment at the site ranged from <0.2 mg/kg to 22.8 mg/kg with a median of 7.3 mg/kg, and all but 10 samples (91%) were below sediment guidelines of 9.79 mg/kg and the Van Gosen [15] median value of <10 mg/kg. Suspended sediment samples at the site had a median of 3.0 mg/kg uranium and 0.14 mg/kg cadmium, with no samples above sediment guidelines of 104.4 mg/kg and 0.99 mg/kg, respectively. Median uranium and cadmium concentrations in suspended sediment are comparable with reported median values for Colorado Plateau surface soils of 3.5 mg/kg and <0.5 mg/kg, respectively [15]. Although evaporation of sample water would not be expected to affect comingled suspended-sediment concentrations, if partitioning were occurring from water to suspended sediment in autosampler bottles, greater element mass would be measured on suspended sediment samples than would have been present in the stream at time of sample collection. Increasing suspended sediment concentration with decreasing aqueous concentrations, however, is not evident in co-collected sample results from the site (Fig 9A).

#### Kanab Creek monitoring site

Fewer runoff events during the 2015–2018 time period of this study resulted in fewer water and suspended sediment samples collected from the Kanab Creek monitoring site as compared with the Little Colorado River site (Table 3; Fig 5B). During flow conditions ranging from baseflow to 780 times daily mean flow at the site, 57 water samples and 23 suspended-sediment samples were collected at Kanab Creek during the study period. A majority of water samples had concentrations below reporting limits for arsenic (63%), cadmium (75%), and lead (74%), with all uranium results above reporting limits (Fig 6B; S8 Table in S1 File). Uranium in highly mobile carbonate complexes is expected to remain in solution at the neutral to slightly alkaline pH and oxic conditions found at the site [48]. Arsenic concentrations in water ranged from <1.5 to 4.5 μg/L with a median of 1.7 μg/L, cadmium concentrations from <0.06 to 0.9 μg/L with a median of 0.03 μg/L, and lead concentrations from <0.2 to 1.5 μg/L with a median of 0.07 μg/L. All arsenic, cadmium, and lead concentrations in water were well below MCL values of 10 μg/L, 5 μg/L, and 15 μg/L, respectively. For these three elements, only a single sample of cadmium (0.9 μg/L) exceeded a chronic exposure aquatic life criteria benchmark (S8 Table in S1 File; Table 2). Uranium concentrations in water in Kanab Creek ranged from 0.8 to 6.4 μg/L with a median value of 2.7 μg/L, substantially below the MCL of 30 μg/L and the chronic exposure aquatic life criteria of 15 μg/L.

Potential increase in sample concentration from evaporation was estimated to be as high as 17% in Kanab Creek samples (S8 Table in S1 File). As with results from the Little Colorado River, increasing environmental sample concentrations is not significantly correlated with increasing amount of time from sample collection to sample retrieval at Havasu Creek (S14 Fig in S1 File). As described previously, evaporation would serve to increase aqueous concentrations, resulting in measured concentrations from autosamplers being higher than what would have been present in the creek at the time of sampling. Only a single pre-and-post deployment comparison out of 20 such comparisons indicated a decrease in aqueous QC concentrations during a deployment (a decrease of 0.5 μg/L of uranium). Of the 57 water samples collected, 21 of them (37%) were processed onsite (Fig 7). Median concentrations of arsenic, cadmium, and lead were similar for onsite and autosampler-collected samples (1.2 versus 2.0 μg/L, 0.05 versus 0.01 μg/L, 0.1 versus 0.04 μg/L, respectively). Uranium concentrations were substantially different between the two types of samples, with a median concentration of 5.4 μg/L for onsite-processed samples and 2.0 μg/L for autosampler-collected samples. This difference could be a result of a small amount of uranium partitioning from aqueous to solid phases in autosampler bottles, although this is not evident in co-collected sample results (Fig 9B), and not expected from pH and redox conditions at the site [48]. It also could be a result of lower uranium concentrations at higher flows that are typically sampled only by autosampler.

Suspended sediment was sampled 23 times during runoff events at Kanab Creek during the 2015–2018 time period of this study (S8 Table in S1 File; Fig 8B). Concentrations for all focus elements were above reporting limits for all samples. Like suspended-sediment samples from the Little Colorado River monitoring site, lead was the most abundant of the focus elements, with a median concentration of 14.2 mg/kg and a maximum concentration of 20 mg/kg, both well below sediment quality guidelines of 35.8 mg/kg (Table 2). Lead in solution is likely to sorb to particulate matter at the pH and redox conditions found at the site [46]. The median lead concentration in suspended sediment at Kanab Creek is comparable to the median value for surface soils on the Colorado Plateau of 13 mg/kg [15]. Arsenic is the next most abundant focus element in Kanab Creek suspended sediment, with a median concentration of 8.5 mg/kg and a maximum concentration of 21 mg/kg. Eight of the 23 suspended sediment samples (35%) exceeded the sediment quality guidelines for arsenic concentrations (9.79 mg/kg; Table 2). Cadmium and uranium concentrations in suspended sediment at the site were both relatively low, with median/maximum concentrations of 0.3/0.5 mg/kg and 2.8/3.4 mg/kg, respectively (S8 Table in S1 File). All cadmium and uranium results were below sediment quality guidelines, and median values were comparable to published median values for Colorado Plateau surface soils of <0.5 mg/kg and 3.5 mg/kg, respectively [15].

#### Havasu Creek monitoring site

There were 38 water samples and 17 suspended-sediment samples collected from the Havasu Creek monitoring site during the 2015–2018 time period of this study (Table 3; Fig 5C). Similar to the other tributary and main-stem monitoring sites, cadmium and lead concentrations were mostly below reporting limits (S8 Table in S1 File). Nineteen of the water samples (50%) had cadmium concentrations that were less than reporting limits and the median value for all cadmium samples was 0.02 μg/L. The maximum observed cadmium concentration of 0.9 μg/L was well below the MCL of 5 μg/L and below the chronic exposure aquatic life criteria of 1.6 μg/L (Table 2). Lead concentrations in water were less than reporting limits for 27 samples (71%), with a median value of 0.02 μg/L. Low concentrations of dissolved cadmium and lead are expected at the neutral to slightly alkaline pH and oxic conditions at the Havasu Creek monitoring site [46, 47]. As described in the QC results section, the maximum lead concentration of 3.8 μg/L in Havasu Creek is potentially affected by contamination, but even so, it is still well below the MCL of 15 and the chronic exposure aquatic life criteria of 7.6 μg/L. Arsenic concentrations in water at Havasu Creek are mostly higher than at any of the other main-stem or tributary monitoring sites in this study (S8 Table in S1 File). All arsenic samples had above-reporting-limits concentrations and ranged from a low of 4.3 μg/L to a high of 16.2 μg/L with a median of 11.3 μg/L. Concentrations of arsenic in 24 of 38 (63%) water samples exceeded the MCL of 10 μg/L. Uranium concentrations in water samples from Havasu Creek ranged from 0.6 to 3.9 μg/L, with a median of 3.2 μg/L.

Potential effects of evaporation on autosampler concentrations at Havasu Creek were estimated to be as high at 34% for a sample collected by autosampler on April 17, 2016 that remained in the sampler until the next retrieval 132 days later. Results for focus elements in this environmental sample, however, were not abnormally high, with concentrations of 11, <0.02, 0.57, and 3.9 μg/L for arsenic, cadmium, lead, and uranium, respectively, highlighting the challenge in quantifying bottle effects in autosamplers (S8 Table in S1 File). As with results from the Little Colorado River and Kanab Creek sites, increasing sample concentrations from Havasu Creek do not significantly correlate with increasing time that samples remained in the autosampler before retrieval (S14 Fig in S1 File). Five pre- and post-deployment Havasu Creek samples indicated declining concentrations during the deployment, with a maximum decline of 1.9 μg/L in a uranium sample (S4 Table in S1 File, S13 Fig in S1 File). While some change in concentrations in water samples may have occurred in autosampler bottles before retrieval due to evaporation and/or partitioning, it is unlikely this caused the elevated dissolved arsenic concentrations observed at the site. ISCO autosampler bottles remain uncapped during their deployment. Access to atmospheric oxygen and limited dissolved organic carbon [26] likely ensure oxic conditions are maintained in the sample bottles. The divalent arsenate anion HAsO_4_^2-^ is expected to predominate in the samples, which will partition to mineral surfaces, resulting in a decrease, not increase, in dissolved concentrations [43]. Arsenic results from samples processed onsite (16 samples) and unaffected by sample bottle processes are comparable to autosampler-collected (22 samples) results, with median values of 11.7 and 8.3 μg/L, respectively (Fig 7). Additionally, a water sample collected by USGS from Havasu Spring, which provides most of the non-runoff flow in Havasu Creek, in October 2016 contained 14 μg/L of arsenic [26]. Likewise, uranium concentrations from samples processed onsite also are similar to autosampler-collected samples, with median values of 3.4 and 2.5 μg/L, respectively; both comparable to the 2016 Havasu Spring result of 3.6 μg/L (Fig 7) [26]. Finally, lower arsenic and uranium concentrations in suspended sediment do not correlate with higher aqueous concentrations in co-collected samples, as might be expected if significant partitioning from suspended sediment to water were occurring (Fig 9C).

Also distinct among the tributary monitoring sites, arsenic is the most abundant element in Havasu Creek suspended sediment (S8 Table in S1 File; Fig 8C). As described previously, the divalent arsenate anion HAsO_4_^2-^ may strongly sorb to mineral surfaces [43]. Arsenic concentrations in Havasu Creek suspended sediment showed substantial variability and ranged from 4.1 to 39.2 mg/kg with a median of 9.4 mg/kg. Six of 17 samples (35%) exceeded sediment quality guidelines for arsenic (Table 2). The highest arsenic concentrations in suspended sediment were from high-flow events in August and September of 2016, when the average concentration was 30.3 mg/kg for the 5 suspended sediment samples collected during that time (Fig 8C). Average arsenic concentration for the remaining 12 suspended sediment samples was only 7.1 mg/kg, more comparable to the median concentration of <10 μg/L reported by Van Gosen [15]. The higher arsenic concentrations were not associated with particularly high flow rates at the site and, owing to limited suspended sediment data at the site, it is unknown how common these higher concentrations may be. The next most abundant focus element in suspended sediment at Havasu Creek was lead, with concentrations from 2.9 to 12.3 mg/kg and a median of 7.2 mg/kg. All suspended sediment samples were substantially lower in lead concentrations than the sediment quality guideline of 35.8 mg/kg and lower than the median value reported for Coconino Plateau soils of 13 mg/kg [15]. Both uranium and cadmium concentrations were relatively low in Havasu Creek suspended sediment, with median values of 2.15 and 0.25 mg/kg, respectively.

#### Comparison among Colorado River tributaries

**Fig 10:**
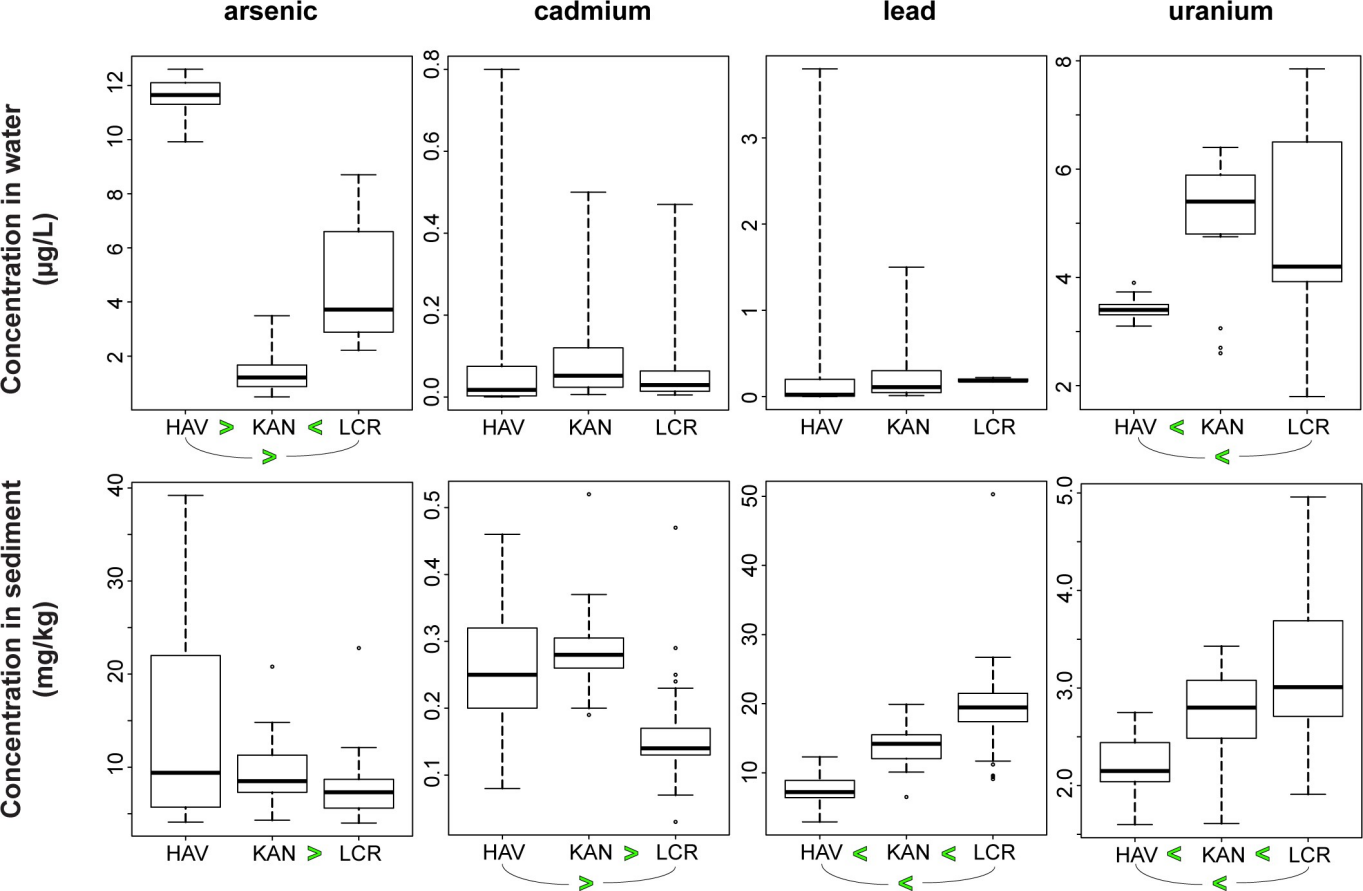
Boxplots showing the distribution of concentrations of arsenic, cadmium, lead, and uranium in water (top row) and suspended sediment (bottom row) at the Little Colorado River (LCR), Kanab Creek (KAN), and Havasu Creek (HAV) tributary sites. Less than (<) and greater than (>) symbols denote statistically significant differences between results from the sites.

Although autosampler-collected water samples allow for monitoring at times when field personnel are not present, particularly during high-flow events, the uncertain effects of evaporation and partitioning in sample bottles that remain onsite until sample retrieval makes statistical comparison of results between monitoring sites problematic. This is less of an issue for suspended sediment in autosampler bottles because evaporation of water from the sample likely has a limited effect on suspended sediment concentrations and small amounts of sorption from the aqueous phase to suspended sediment would not greatly alter solid phase elemental mass. To compare focus element concentrations in water from Colorado River tributaries that were monitored for this study, only results from water samples that were processed onsite are used. Havasu Creek has significantly (and substantially) higher arsenic concentrations in water samples than either Kanab Creek or Little Colorado River, but also significantly lower uranium concentrations in water than either of the other tributary sites (Fig 10). Little Colorado River has significantly higher arsenic concentrations in water than Kanab Creek (Fig 10). No significant difference is seen in cadmium or lead concentrations in water between the three tributary sites. Little Colorado River suspended sediment is significantly higher in both lead and uranium than both Havasu Creek and Kanab Creek, but is significantly lower in cadmium than both sites (Fig 10). Little difference is observed in arsenic concentrations in suspended sediment, with the only statistically significant difference that Little Colorado River suspended sediment has slightly less arsenic than Kanab Creek.

#### Estimates of focus-element loads entering the Colorado River from major tributaries

**Fig 11:**
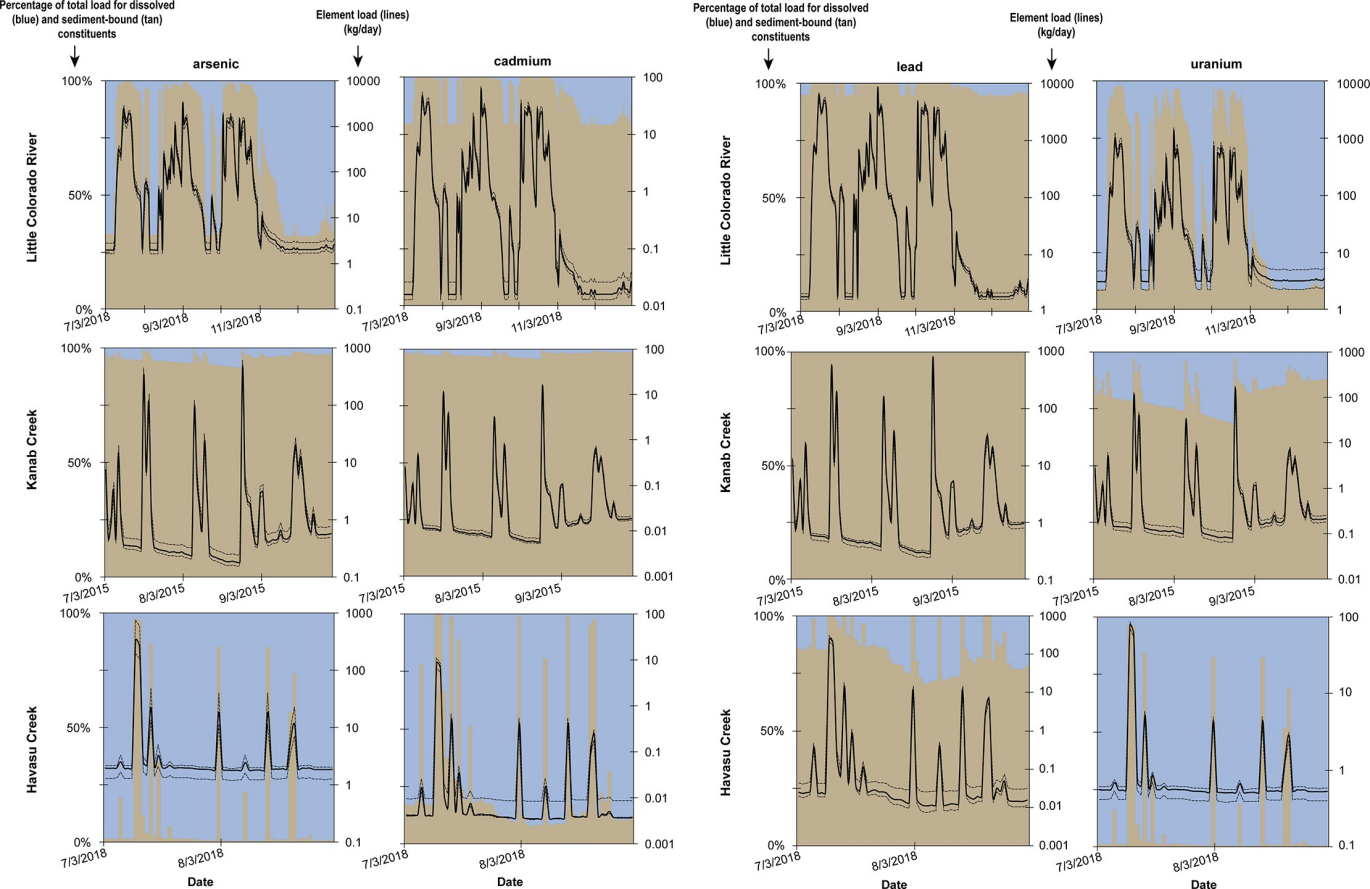
Dissolved and suspended-sediment-bound percentage, and estimates of the total load of arsenic, cadmium, lead, and uranium from the Little Colorado River, Kanab Creek, and Havasu Creek tributary sites during select time periods. Blue and tan areas represent dissolved and suspended-sediment-associated percentages (left axis), respectively. Solid line is total load (right axis) estimated with median concentration values while dashed lines used interquartile range of concentrations to illustrate variance.

The loading of arsenic, cadmium, lead, and uranium entering the Colorado River from Little Colorado River, Kanab Creek, and Havasu Creek was estimated using streamflow data, sediment discharge estimates, water quality data, and suspended sediment quality data. Loading was estimated only for select high- and base-flow time periods to illustrate the variability in metal loading entering the Colorado River from these tributaries (S15 Fig in S1 File). Median concentration values for water and suspended sediment were used for load estimates, with interquartile range estimates provided to illustrate potential variance in load related to concentration. Temporal trace element loads from the tributary sites are greatest in the highest flow Little Colorado River during both baseflow and runoff conditions (Fig 11). The pattern of loading from the Little Colorado River is dominated by irregular suspended sediment discharge occurring during flood events at the site, resulting in loads that can range over 3 orders of magnitude for some elements (Fig 11). During baseflow conditions, dissolved arsenic makes up about 67% of the ~2 kg/d of total arsenic loading from the Little Colorado River (Fig 11). During runoff events, however, suspended-sediment-bound arsenic makes up over 99% of the total arsenic load, which reaches 2900 kg/d during the high flow period in Fig 11. Similarly, the load of uranium during baseflow conditions in the Little Colorado River is dominated by the dissolved component, with about 91% of the total load of ~2 kg/d from dissolved uranium (Fig 11). About 97% of the total uranium load in the Little Colorado River during runoff conditions is from the suspended-sediment-bound portion, which can be >1000 kg/d during some flood events. Owing to the substantially higher concentrations of both cadmium and lead associated with suspended sediment than dissolved in water, the suspended-sediment-bound portion dominates loading during both baseflow and runoff conditions (Fig 11). Suspended sediment composes 80% of the 0.02 kg/d of cadmium and 95% of the ~2 kg/d of lead during baseflow conditions at the Little Colorado River monitoring site. During runoff conditions, more than 99% of the cadmium and lead loading is associated with suspended sediment, with as much as 56 kg/d of total cadmium and >7700 kg/d of total lead entering the Colorado River from the Little Colorado River tributary during high flows of limited duration (Fig 11).

The pattern of loading from Kanab Creek also is dominated by irregular suspended sediment discharge occurring during flood events at the site, with loads ranging over 3+ orders of magnitude for all elements (Fig 11). At the Kanab Creek monitoring site during baseflow conditions, the loading associated with suspended sediment makes up over 93% of the total load of arsenic, cadmium, and lead, and over 75% of the uranium load (Fig 11). Baseflow loading of arsenic, cadmium, lead, and uranium at Kanab Creek are all <0.4 kg/d. Runoff conditions increase the suspended sediment portion of total loading to >99% for arsenic, cadmium, and lead, and 97% of uranium, with loading reaching as high as ~470 kg/d, ~15 kg/d, ~780 kg/d, and ~160 kg/d for arsenic, cadmium, lead, and uranium, respectively, during short duration high-flow events (Fig 11).

At the Havasu Creek monitoring site, the loading of most metals can vary within 2 orders of magnitude between baseflow and runoff conditions except for lead, which can vary by a factor of >22000 between low and high flows (Fig 11). In contrast to loads from Kanab Creek, loads from Havasu Creek are dominated by dissolved metals during baseflow conditions for arsenic (99% of ~2 kg/d), cadmium (>80% of ~0.004 kg/d), and uranium (99% of ~0.56 kg/d). Dissolved lead makes up about 15–20% of ~0.03 kg/d of total lead loading during baseflow conditions at the site. Brief high-flow episodes may contribute as much as 330 kg/d of arsenic, 9.6 kg/d of cadmium, 247 kg/d of lead, and 76 kg/d of uranium to the Colorado River from Havasu Creek, over 97% of which is associated with suspended sediment.

While the loading of some metals from tributaries to the Colorado River in Grand Canyon can be substantial during brief high-flow events, the relative difference in streamflow between the tributaries and main-stem river means that, at current observed concentrations, little effect on dissolved Colorado River concentrations is expected. For example, using daily mean flow and mean observed concentrations on main-stem sites, concentration of dissolved uranium from all tributaries would need to be 38 μg/L consistently to increase the uranium concentration at Colorado River–Diamond Creek by just 1 μg/L from 3.4 to 4.4 μg/L. Tributary uranium concentrations would need to be 800 μg/L consistently to exceed the uranium MCL of 30 μg/L at the Diamond Creek main-stem site. By comparison, the highest median dissolved uranium concentration at any of the tributary sites was 5.4 μg/L during this study. At the concentrations observed during this study, tributaries contributed an estimated 0.12 μg/L of arsenic and 0.03 μg/L of uranium to the main-stem river.

## Summary and conclusions

In 2015–2018, major surface waters in Grand Canyon were monitored for select metals associated with mineralized uranium deposits in the area, including uranium, arsenic, cadmium, and lead. Dissolved constituents in the main-stem Colorado River were monitored upstream (Lees Ferry), in the middle (Phantom Ranch), and downstream (Diamond Creek) of Grand Canyon National Park and breccia-pipe uranium mining areas. Cadmium and lead concentrations were mostly below reporting limits at the main-stem sites, while arsenic concentrations were 1–2 μg/L and uranium concentrations were 2.5–4 μg/L. Dissolved and suspended-sediment-bound constituents were monitored at the mouths of the Little Colorado River, Kanab Creek, and Havasu Creek tributaries, whose watersheds have experienced different levels of uranium mining activities over time. Median and 75^th^ percentiles of dissolved cadmium and lead concentrations were ≤0.3 μg/L at all tributary sites and median and 75^th^ percentiles of dissolved uranium concentrations at the tributary sites were ≤8.3 μg/L. Only dissolved arsenic concentrations at Havasu Creek, with median and 75^th^ percentiles of concentrations of 11.3 and 12.1 μg/L, respectively, were regularly above MCL benchmarks. Suspended-sediment concentrations for all focus elements were mostly below sediment quality guidelines with the exception of a single lead sample at Little Colorado River and arsenic concentrations in 9%, 35%, and 35% of suspended sediment samples from Little Colorado River, Kanab Creek, and Havasu Creek, respectively.

Autosampler-collected water samples allow for monitoring at times when field personnel are not present at the tributary sites; however, potential changes in concentrations in autosampler bottles that remain onsite until sample retrieval and processing makes statistical comparison of results between monitoring sites problematic. Comparison of focus element concentrations among tributary sites using only onsite processed samples indicated statistically significant higher dissolved arsenic concentrations at Havasu Creek than either of the other tributary sites. Little Colorado River and Kanab Creek both had significantly higher dissolved uranium concentrations than Havasu Creek. Uranium and lead concentrations in suspended sediment were significantly higher in the Little Colorado River than in either Kanab Creek or Havasu Creek. Focus element loading from the tributary sites is dominated by irregular suspended sediment discharge occurring during flood events at the sites, resulting in loads that can range over 3+ orders of magnitude between baseflow and high flows for some elements. In the tributary sites monitored, most focus-element mass enters the Colorado River from the highest flow Little Colorado River, including loads as high as 2900 kg/d of arsenic, 56 kg/d of cadmium, 7700 kg/d of lead, and 1200 kg/d of uranium during short duration runoff events. Over 97% of the load during the flood events is associated with suspended sediment at the Little Colorado River monitoring site. While the loading of some metals from tributaries to the Colorado River in Grand Canyon can be substantial at times, the relative difference in streamflow between the tributaries and main-stem river results in little effect of observed tributary concentrations on dissolved Colorado River concentrations. At the concentrations observed during this study, tributaries contributed on average only about 0.12 μg/L of arsenic and 0.03 μg/L of uranium to the main-stem river. This study demonstrates how chemical loading from mined watersheds may be reliably assessed across a wide range of flow conditions in a challenging environment.

## Supporting information

S1 File (PDF)

S1 Data Water and suspended sediment chemistry results for all monitoring sites. (XLSX)
