# Supplementary material for: Assessing uranium and select trace elements associated with breccia pipe uranium deposits in the Colorado River and main tributaries in Grand Canyon, USA
Source: PLoS One. 2020 Nov 4;15(11):e0241502. doi: 10.1371/journal.pone.0241502 (PMC7641433; doi:10.1371/journal.pone.0241502)
Supplement: S1 File — (PDF) [file pone.0241502.s001.pdf]

PLOS ONE

Supporting Information for

Assessing uranium and select trace elements associated with breccia pipe uranium deposits in the Colorado River and main tributaries in Grand Canyon, USA

Fred D Tillman[1]*, Jessica R. Anderson[2], Joel A. Unema[2], Thomas P. Chapin[3]

[1] U.S. Geological Survey, Arizona Water Science Center, Tucson, Arizona, United States of America
[2] U.S. Geological Survey, Arizona Water Science Center, Flagstaff, Arizona, United States of America
[3] U.S. Geological Survey; Geology, Geophysics and Geochemistry Science Center; Denver, Colorado, United States of America

* Corresponding author
E-mail: ftillman@usgs.gov

This Supporting Information provides additional material relevant to the investigation of uranium and select trace elements associated with breccia pipe uranium deposits in the Colorado River and main tributaries in Grand Canyon, USA. This material includes:

- Photographs of U.S. Geological Survey (USGS) stream and river monitoring sites referred to in the main text (**S1–S10 Figs**).
- Results from quality assurance evaluation of the USGS Geology, Geophysics, and Geochemistry Science Center laboratory (**S1 Table**).
- Results from quality control samples at the Little Colorado River, Kanab Creek, and Havasu Creek monitoring sites (**S2–S4 Tables; S11–S13 Figs**).
- A description of laboratory experiments designed to assess the potential partitioning of uranium and associated trace elements between suspended sediment and water in autosampler bottles (**S5–S7 Tables**).
- Tributary sample concentrations plotted against the amount of time between sample collection and sample retrieval (**S14 Fig**).
- Streamflow discharge and sediment discharge data for the Little Colorado River, Kanab Creek, and Havasu Creek monitoring sites during the 2015–2018 study period (**S15 Fig**).
- Excel file of water and sediment results for all monitoring sites (**S8 Table** – separate file). Data presented in the Excel file also are available at USGS ScienceBase (https://doi.org/10.5066/P9RTT4AB).

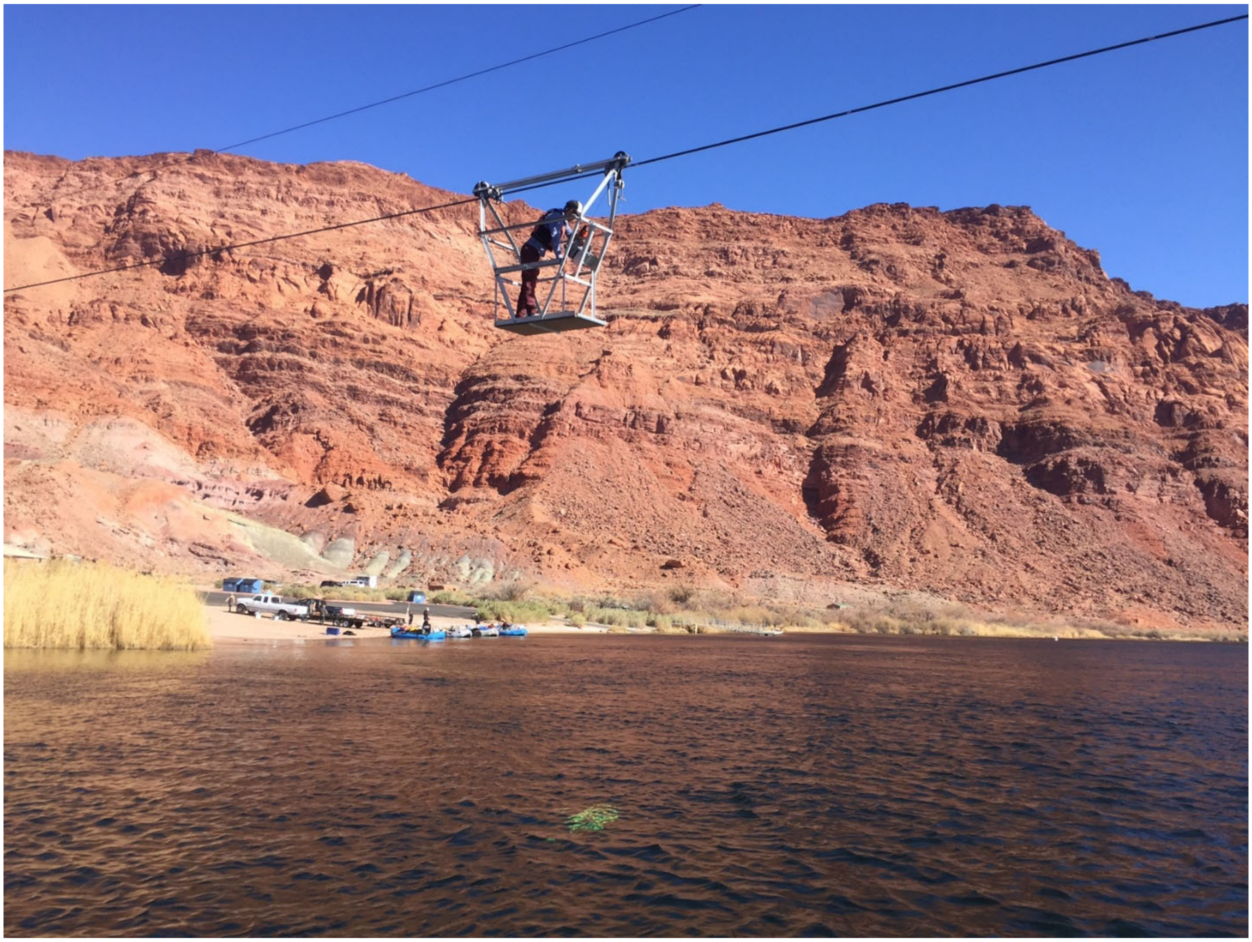

S1 Fig. Water samples being collected from a cableway at monitoring site Colorado River at Lees Ferry, Arizona (USGS site ID 09380000). Photo taken in February 2018 by Joel Unema (USGS).

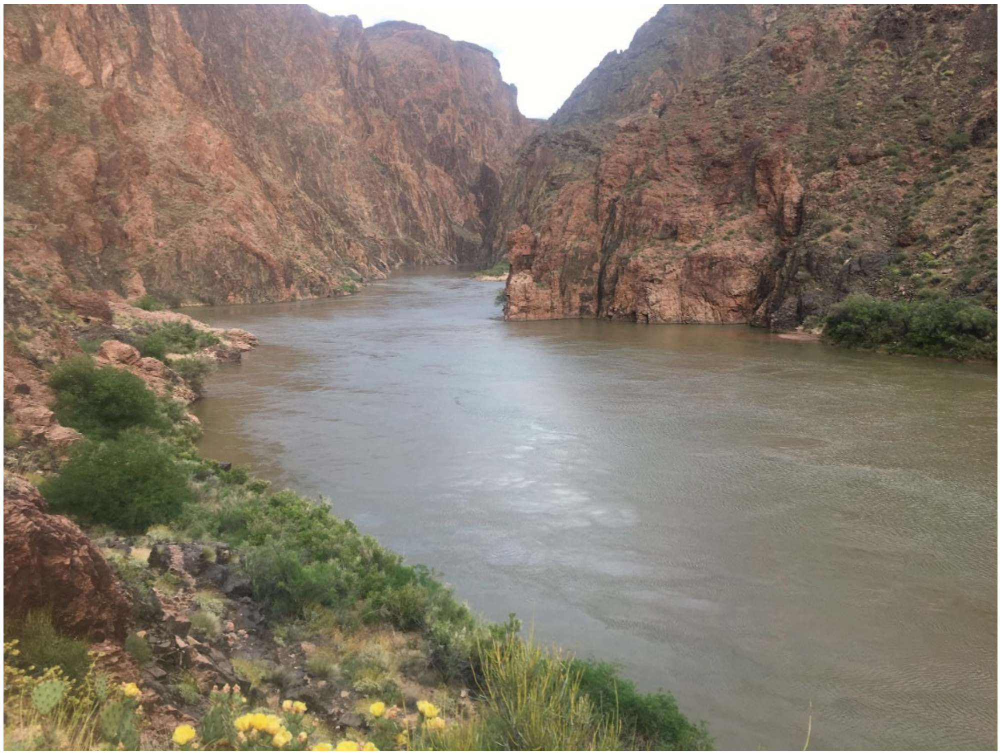

S2 Fig. Monitoring site Colorado River near Grand Canyon (USGS site ID 09402500). Photo taken in April 2019 by Jessica Anderson (USGS).

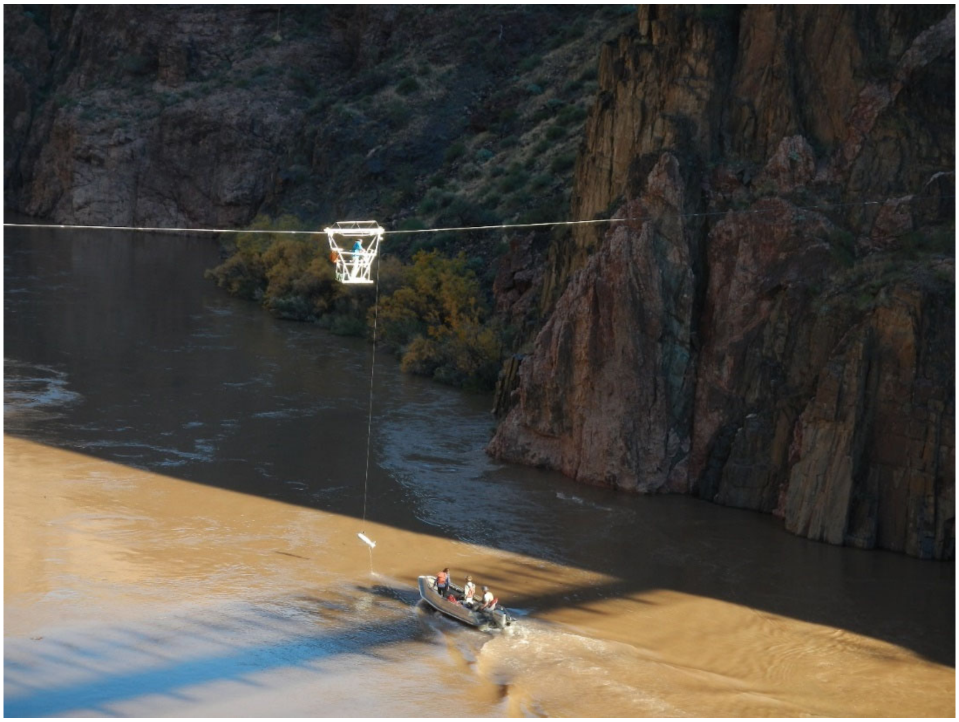

S3 Fig. Water samples being collected at monitoring site Colorado River near Grand Canyon (USGS site ID 09402500). Photo taken in November 2013 by Jessica Anderson (USGS).

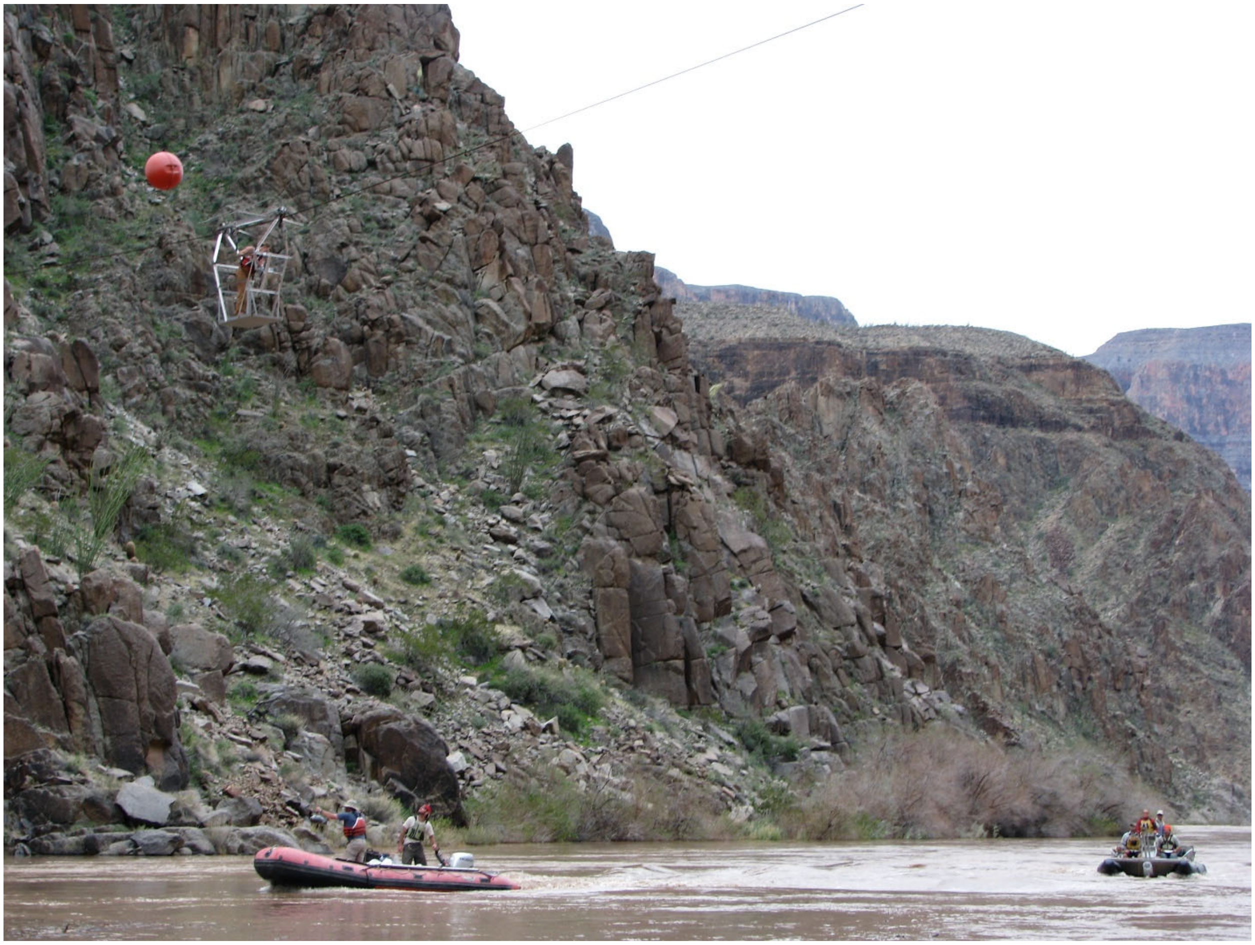

S4 Fig. Water samples being collected at monitoring site Colorado River above Diamond Creek near Peach Springs, Arizona (USGS site ID 09404200). Photo taken in March 2008 by Jessica Anderson (USGS).

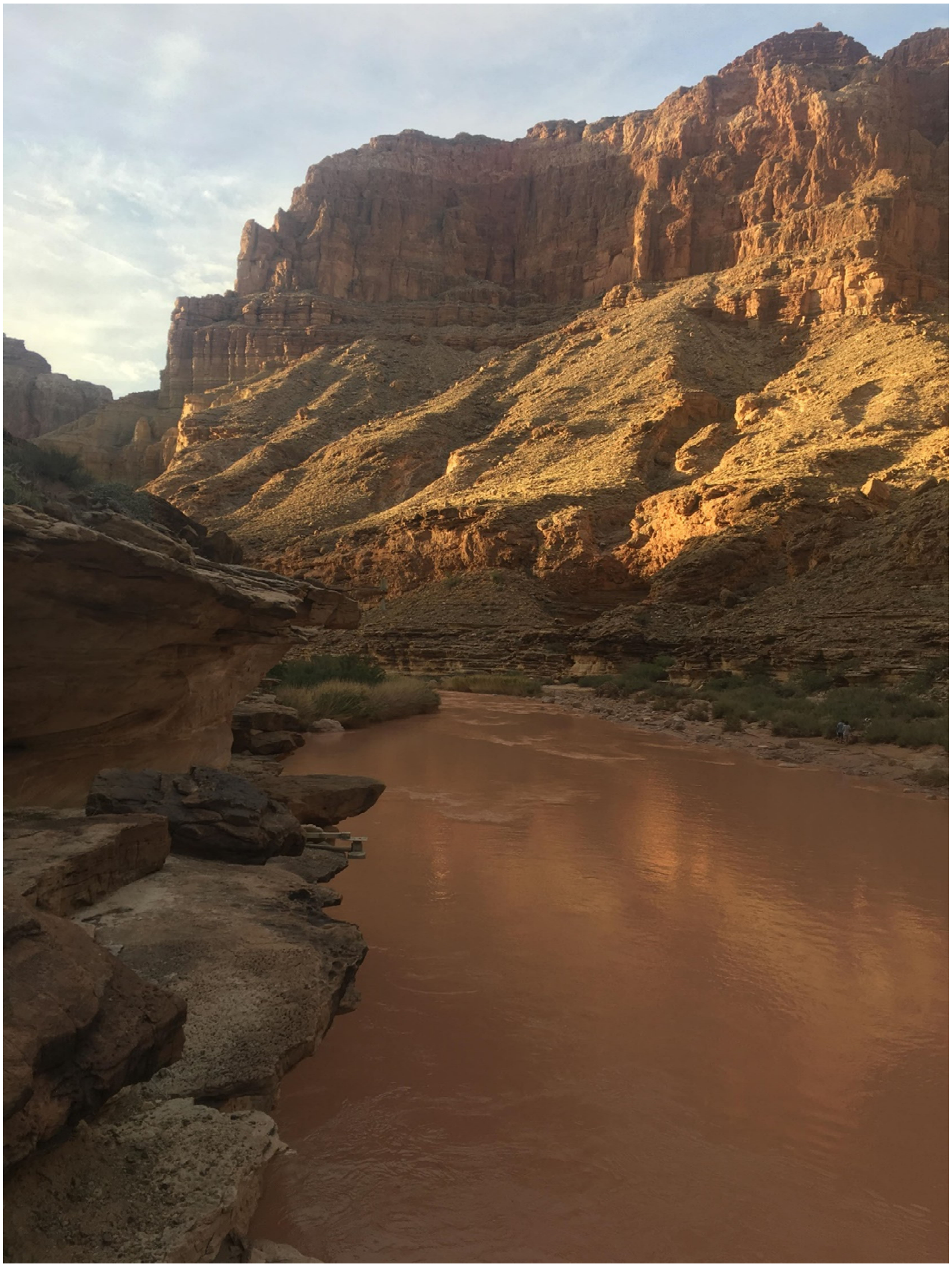

S5 Fig. Monitoring site at Little Colorado River above the mouth near Desert View, Arizona (USGS site ID 09402300). Photo taken in November 2018 by Michael Robinson (USGS).

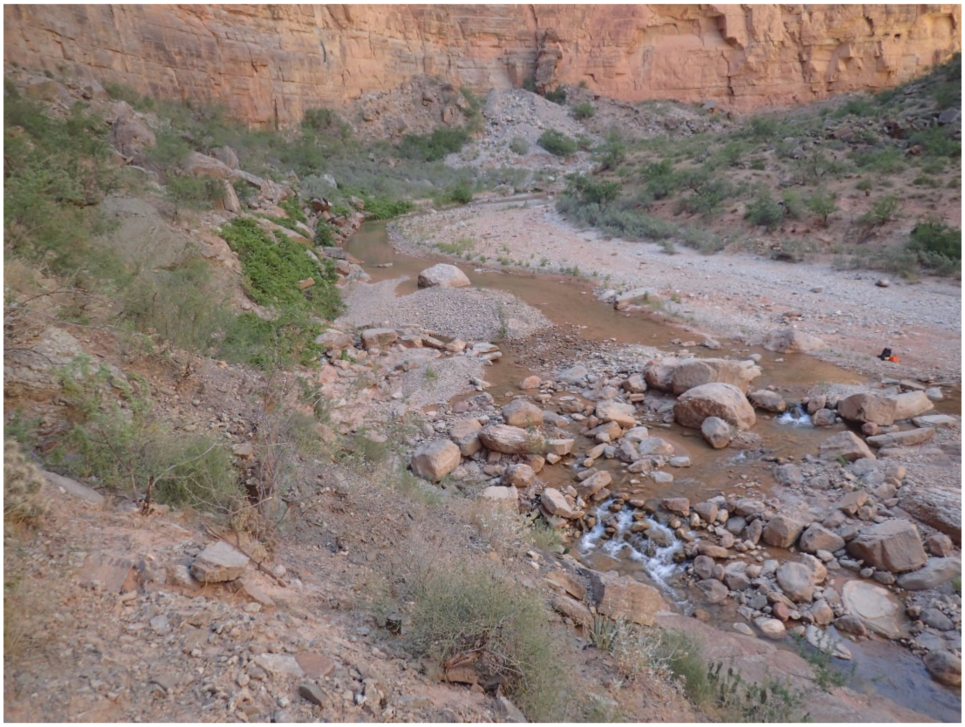

S6 Fig. Monitoring site at Kanab Creek above the mouth near Supai, Arizona (USGS site ID 09403850) during baseflow conditions. Photo taken in November 2018 by Joel Unema (USGS).

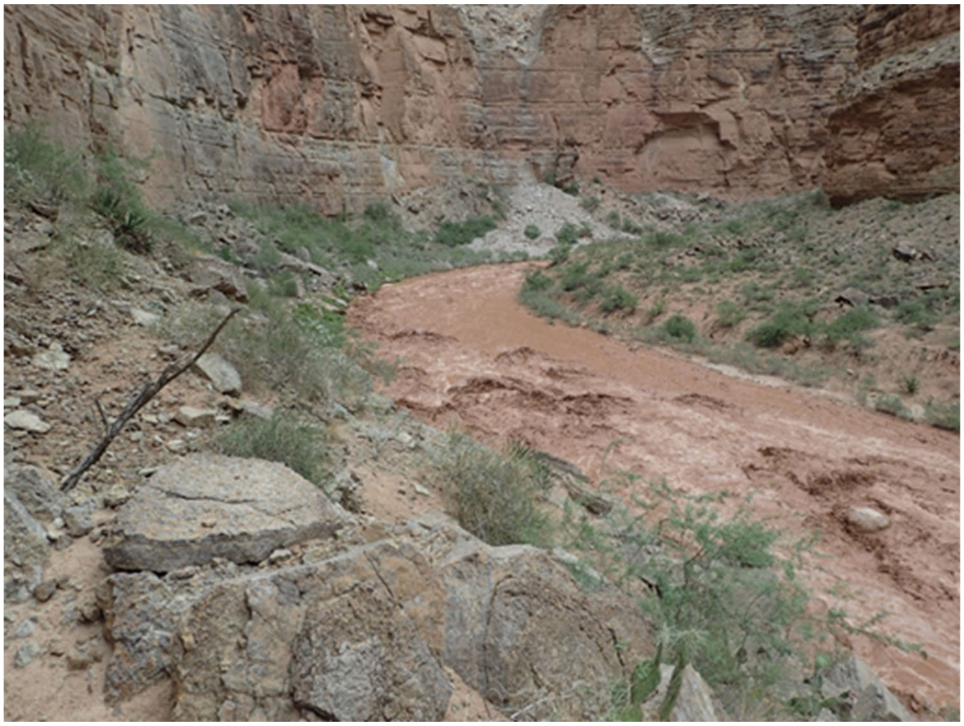

S7 Fig. Monitoring site at Kanab Creek above the mouth near Supai, Arizona (USGS site ID 09403850) during runoff conditions (127 m^3/s). Photo taken on August 23, 2018 by Michael Robinson (USGS).

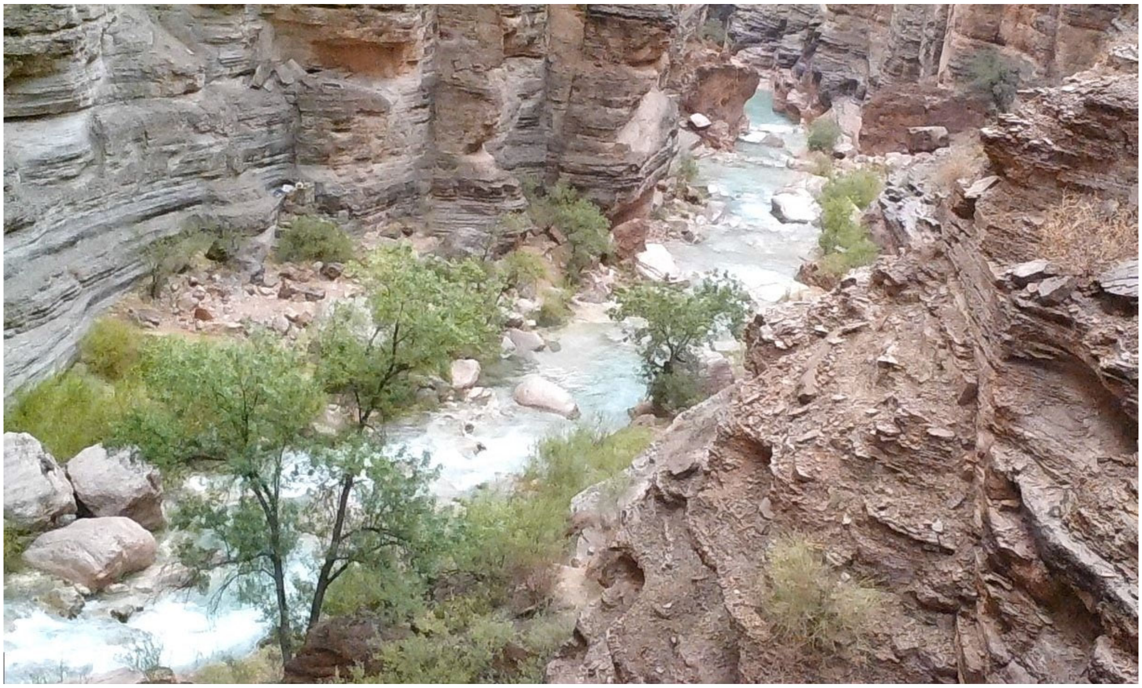

S8 Fig. Monitoring site at Havasu Creek above the mouth near Supai, Arizona (USGS site ID 09404115) during baseflow conditions. Photo taken in July 2018 by Joel Unema (USGS).

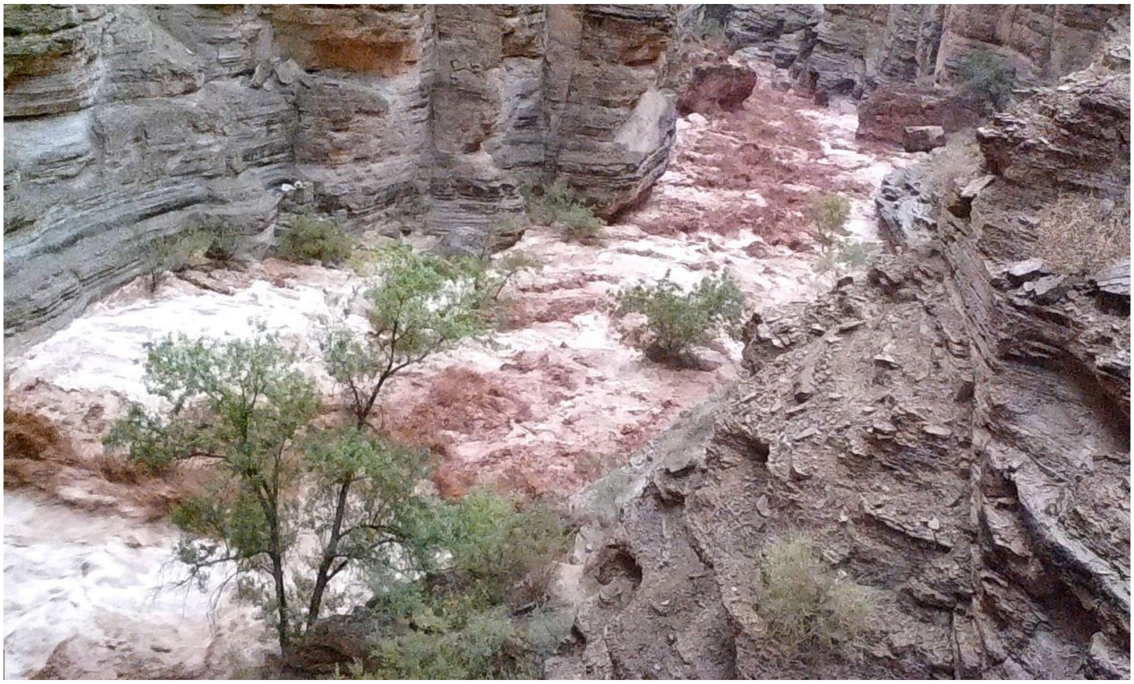

S9 Fig. Monitoring site at Havasu Creek above the mouth near Supai, Arizona (USGS site ID 09404115) during runoff conditions (113 m^3/s). Photo taken in July 2018 by Joel Unema (USGS).

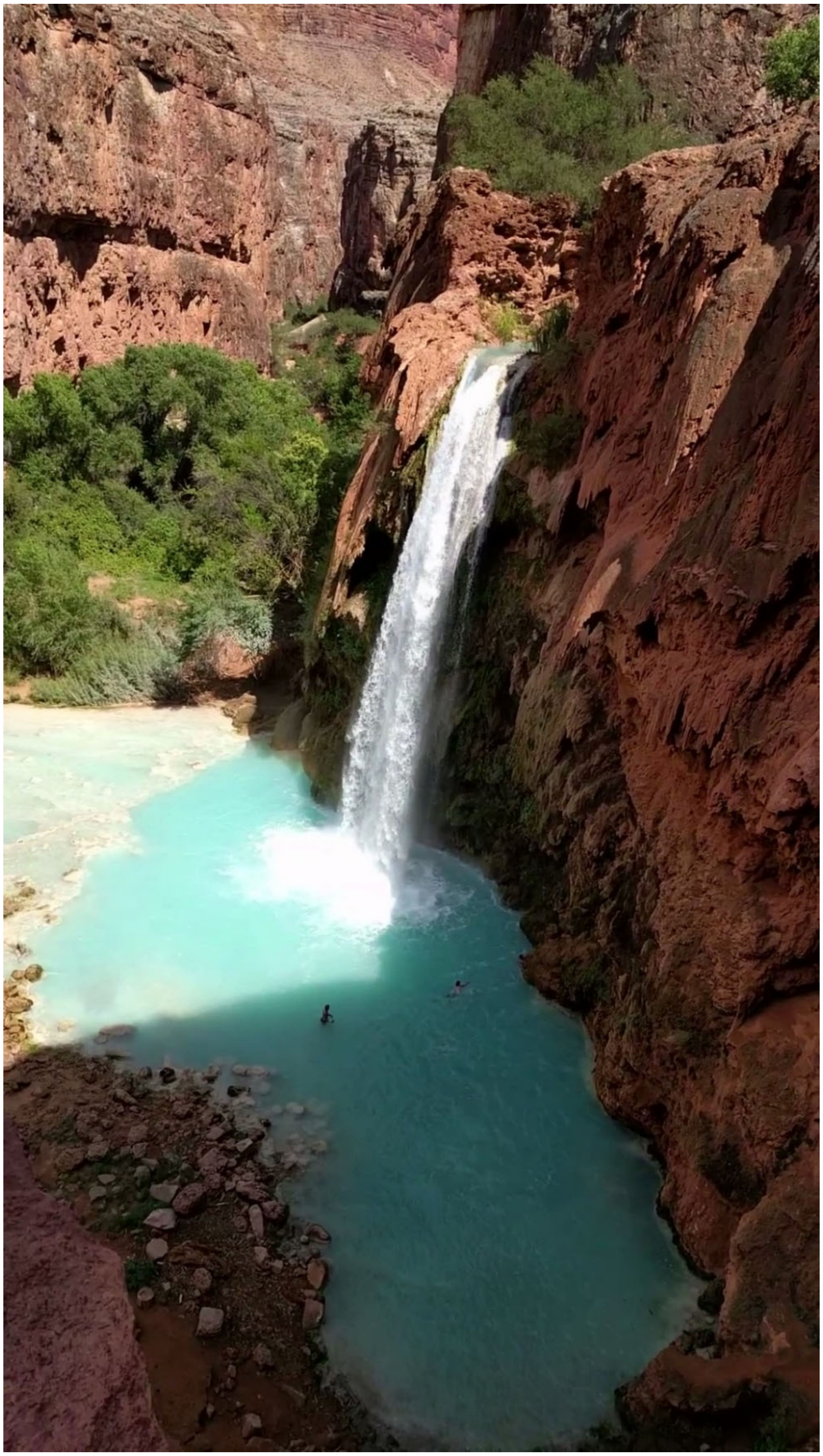

S10 Fig. Havasu Falls on Havasu Creek, approximately 2.5 km downstream from Supai Village and 9 km upstream from monitoring site Havasu Creek above the mouth near Supai, Arizona (USGS site ID 09404115). Photo taken in August 2016 by Fred Tillman (USGS).

S1 Table. Results from quality assurance evaluation of the USGS Geology, Geophysics, and Geochemistry Science Center laboratory.

Evaluation period	Result description[1]	As [µg/L]	Cd [µg/L]	Pb [µg/L]	U [µg/L]
Fall 2016	Reported	0.35	0.56	6.91	0.65
	MPV	0.315	0.478	6.73	0.661
	% difference	10.5%	15.8%	2.6%	1.7%
Spring 2017	Reported	13.3	2.09	13.6	6.59
	MPV	12.8	1.89	13.8	8.31
	% difference	3.8%	10.1%	1.5%	23.1%
Fall 2017	Reported	3.69	0.62	3.8	2.38
	MPV	3.61	0.417	3.71	2.37
	% difference	15.5%	39.2%	2.4%	0.4%
Spring 2018	Reported	3.6	2.6	0.25	1.35
	MPV	4.1	2.83	0.333	1.44
	% difference	13.0%	8.5%	28.5%	6.5%
Fall 2018	Reported	5.3	0.03	1.28	1.65
	MPV	4.8	0.028	1.2	1.51
	% difference	9.9%	6.9%	6.5%	8.9%
Overall average % difference		4.2%	14.1%	6.2%	7.7%

[1]Reported: laboratory reported value; MPV: most probable value based on results from other laboratories; % difference: difference between reported and MPV values divided by their mean.

S2 Table. Quality control water sample results for Little Colorado River monitoring site.
[Rep = replicate, ISCO = autosampler, NA = not available, Ref = reference, As = arsenic, Cd = cadmium, Pb = lead, U = uranium]

Autosampler deployment	Dates	Quality control description	As [µg/L]	Cd [µg/L]	Pb [µg/L]	U [µg/L]
1	2/23/2016 to 4/27/2016	Stream Rep	3.0	0.18	NA	4.0
		ISCO Rep (start)	2.8	0.47	NA	3.0
		ISCO Rep (end)	2.7	<0.06	0.2	3.7
		Blank	<1.5	<0.06	<0.15	<0.15
2	4/27/2016 to 8/21/2016	Stream Rep	8.2	<0.06	0.18	3.9
		ISCO Rep (start)	8.5	<0.06	0.22	4.5
		Ref Sample (start)	7.7	7.6	7.8	6.1
		ISCO Rep (end)	7.9	<0.06	<0.15	1.5
		Ref Sample (end)	8.9	9.2	8.7	8.9
		Blank	<1.5	<0.06	<0.15	<0.15
3	8/21/2016 to 11/5/2016	Blank	<2.5	0.1	<0.25	<0.25
4	11/5/2016 to 2/19/2017	Stream Rep	3.0	<0.08	<0.2	7.3
		ISCO Rep (start)	3.6	<0.08	<0.2	7.9
		Ref Sample (start)	92.8	93.3	92.3	92.6
		ISCO Rep (end)	<2	<0.08	<0.2	9.9
		Ref Sample (end)	90.7	98.4	94.1	92.5
		Blank	<2	<0.08	<0.2	<0.2
5	2/19/2017 To 6/7/2017	Stream Rep	2.7	<0.08	NA	1.8
		ISCO Rep (start)	2.6	<0.08	NA	1.9
		Ref Sample (start)	93.7	94.7	95.3	91.9
		ISCO Rep (end)	2.8	0.4	NA	3.4
		Ref Sample (end)	97.3	99.3	102.7	99.1
		Blank	<2	<0.08	0.3	<0.2
6	6/7/2017 to 8/20/2017	Stream Rep	7.6	<0.08	NA	4.2
		ISCO Rep (start)	7.1	<0.08	NA	3.7
		Ref Sample (start)	87.4	93.4	96.2	93.9
		ISCO Rep (end)	8.0	0.4	NA	1.0
		Ref Sample (end)	94.5	96.5	101.0	97.9
		Blank	<2	<0.08	0.5	<0.2
7	8/20/2017 to 8/20/2018	Stream Rep	<5	<0.2	<0.5	7.8
		ISCO Rep (start)	<5	<0.2	<0.5	7.8
		Ref Sample (start)	101.3	107.3	107.4	101.8
		ISCO Rep (end)	<5	0.3	0.6	21.8
		Ref Sample (end)	225.2	248.0	252.3	235.0
		Blank	<5	0.7	0.9	0.6
8	8/20/2018 to 2/19/2019	Stream Rep	<5	<0.2	<0.5	6.4
		ISCO Rep (start)	<5	<0.2	<0.5	6.6
		Ref Sample (start)	11.9	13.1	13.2	12.4
		ISCO Rep (end)	<5	<0.2	<2	8.4
		Ref Sample (end)	13.5	13.1	12.6	12.3
		Blank	<5	<0.2	<2	<0.5

S3 Table. Quality control water sample results for Kanab Creek monitoring site.
[Rep = replicate, ISCO = autosampler, NA = not available, Ref = reference, As = arsenic, Cd = cadmium, Pb = lead, U = uranium]

Autosampler deployment	Dates	Quality control description	As [µg/L]	Cd [µg/L]	Pb [µg/L]	U [µg/L]
1	8/21/2015 to 2/27/2016	No QC samples deployed				
2	2/27/2016 to 8/25/2016	Stream Rep	<1.5	0.12	0.39	6.0
		ISCO Rep (start)	<1.5	0.34	0.39	6.0
		Ref Sample (start)	8.1	9.6	9.85	7.8
		ISCO Rep (end)	<1.5	<0.06	<0.15	7.8
		Ref Sample (end)	10.7	10.6	10.3	10.5
		Blank	<1.5	<0.06	<0.15	<0.15
3	8/25/2016 to 11/14/2016	Stream Rep	<2	<0.08	<0.2	4.8
		ISCO Rep (start)	<2	<0.08	<0.2	4.8
		ISCO Rep (end)	<2	<0.08	<0.2	4.3
		Blank	<2	0.1	<0.2	<0.2
4	11/14/2016 to 2/24/2017	Stream Rep	<2	0.2	NA	5.6
		ISCO Rep (start)	<2	<0.08	NA	5.3
		Ref Sample (start)	94.8	96.0	94.8	90.9
		ISCO Rep (end)	<2	0.2	NA	5.4
		Ref Sample (end)	93.8	97.3	95.2	90.1
		Blank	NA	NA	NA	NA
5	2/24/2017 to 8/25/2017	Stream Rep	<2	0.4	NA	5.4
		ISCO Rep (start)	<2	0.3	NA	5.5
		Ref Sample (start)	81.3	82.7	86.9	85.7
		ISCO Rep (end)	<2	0.6	NA	6.6
		Ref Sample (end)	101.3	107.2	110.2	100.0
		Blank	<2	<0.08	0.4	<0.2
6	8/25/2017 to 8/24/2018	Stream Rep	2.7	0.5	0.5	6.0
		ISCO Rep (start)	2.3	<0.08	<0.2	5.4
		Ref Sample (start)	108.9	105.4	101.5	106.4
		ISCO Rep (end)	4.4	1.9	1.8	8.0
		Ref Sample (end)	140.7	141.5	142.0	140.2
		Blank	<2	<0.08	<0.2	<0.2
7	8/24/2018 to 2/19/2019	Stream Rep	<2	0.1	<0.2	2.7
		ISCO Rep (start)	<2	<0.08	0.23	2.6

S4 Table. Quality control water sample results for Havasu Creek monitoring site.
[Rep = replicate, ISCO = autosampler, NA = not available, Ref = reference, As = arsenic, Cd = cadmium, Pb = lead, U = uranium]

Autosampler deployment	Dates	Quality control description	As [µg/L]	Cd [µg/L]	Pb [µg/L]	U [µg/L]
1	8/23/2015 to 2/29/2016	No QC samples deployed				
2	2/29/2016 to 8/27/2016	Stream Rep	9.9	<0.1	<0.25	3.3
		ISCO Rep (start)	11.8	0.3	0.5	3.7
		Ref Sample (start)	9.2	8.8	9.9	8.5
		ISCO Rep (end)	19.4	<0.1	<0.25	4.4
		Ref Sample (end)	14.0	13.3	13.2	12.9
		Blank	<2.5	<0.1	<0.25	<0.25
3	8/27/2016 to 11/15/2016	Stream Rep	11.6	<0.1	<0.2	3.3
		ISCO Rep (start)	11.3	<0.1	<0.2	3.3
		ISCO Rep (end)	12.1	<0.1	<0.2	2.0
4	11/15/2016 to 2/25/2017	Stream Rep	11.8	0.1	<0.2	3.4
		ISCO Rep (start)	11.2	<0.08	<0.2	3.1
		Ref Sample (start)	93.0	98.4	94.8	91.1
		ISCO Rep (end)	11.3	<0.08	0.2	3.4
		Ref Sample (end)	92.2	99.6	96.0	91.4
		Blank	<2	<0.08	<0.2	<0.2
5	2/25/2017 to 8/26/2018	Stream Rep	12.6	<0.08	<0.2	3.6
		ISCO Rep (start)	12.1	0.3	0.3	3.9
		Ref Sample (start)	94.9	86.2	90.5	91.0
		ISCO Rep (end)	23.9	6.7	7.6	10.5
		Ref Sample (end)	416.3	402.9	416.1	410.4
6	8/26/2017 to 11/12/2018	Stream Rep	11.6	<0.08	<0.2	3.5
		ISCO Rep (start)	12.5	0.8	3.8	3.5
		Ref Sample (start)	10.4	10.1	10.5	10.8
		ISCO Rep (end)	13.2	0.1	<0.2	1.6
		Ref Sample (end)	11.6	11.7	11.8	11.8
		Blank	<2	<0.08	<0.2	<0.2
7	11/12/2018 to 2/25/2019	Stream Rep	11.3	<0.08	<0.2	3.4
		ISCO Rep (start)	10.5	<0.08	<0.2	3.5

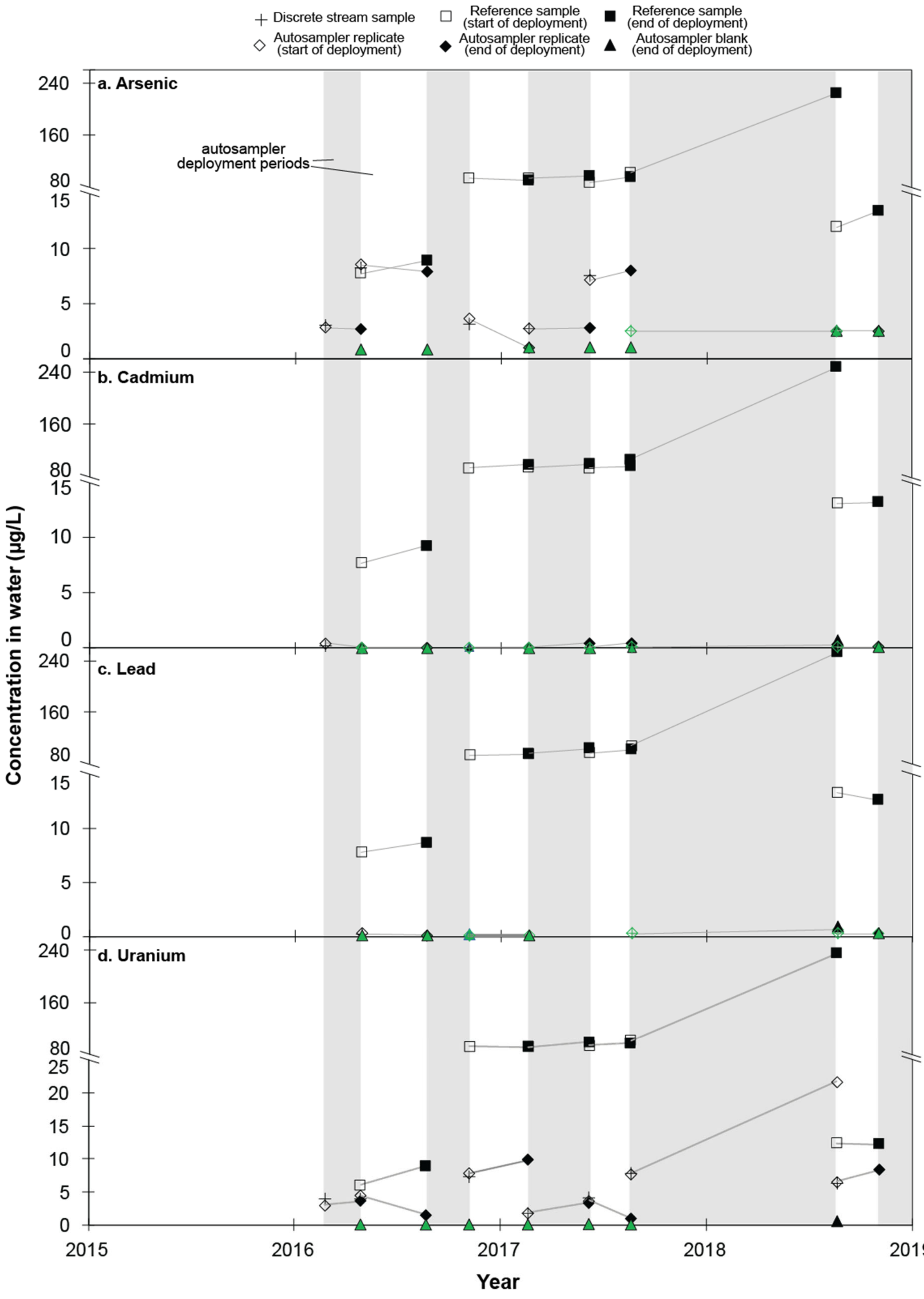

S11 Fig. Results from quality control (QC) samples at the Little Colorado River monitoring site. Symbols in green indicate values below reporting limits and are plotted at one-half the reporting limit. Lines drawn to connect pre- and post-deployment QC sample pairs do not imply a linear concentration change over the deployment.

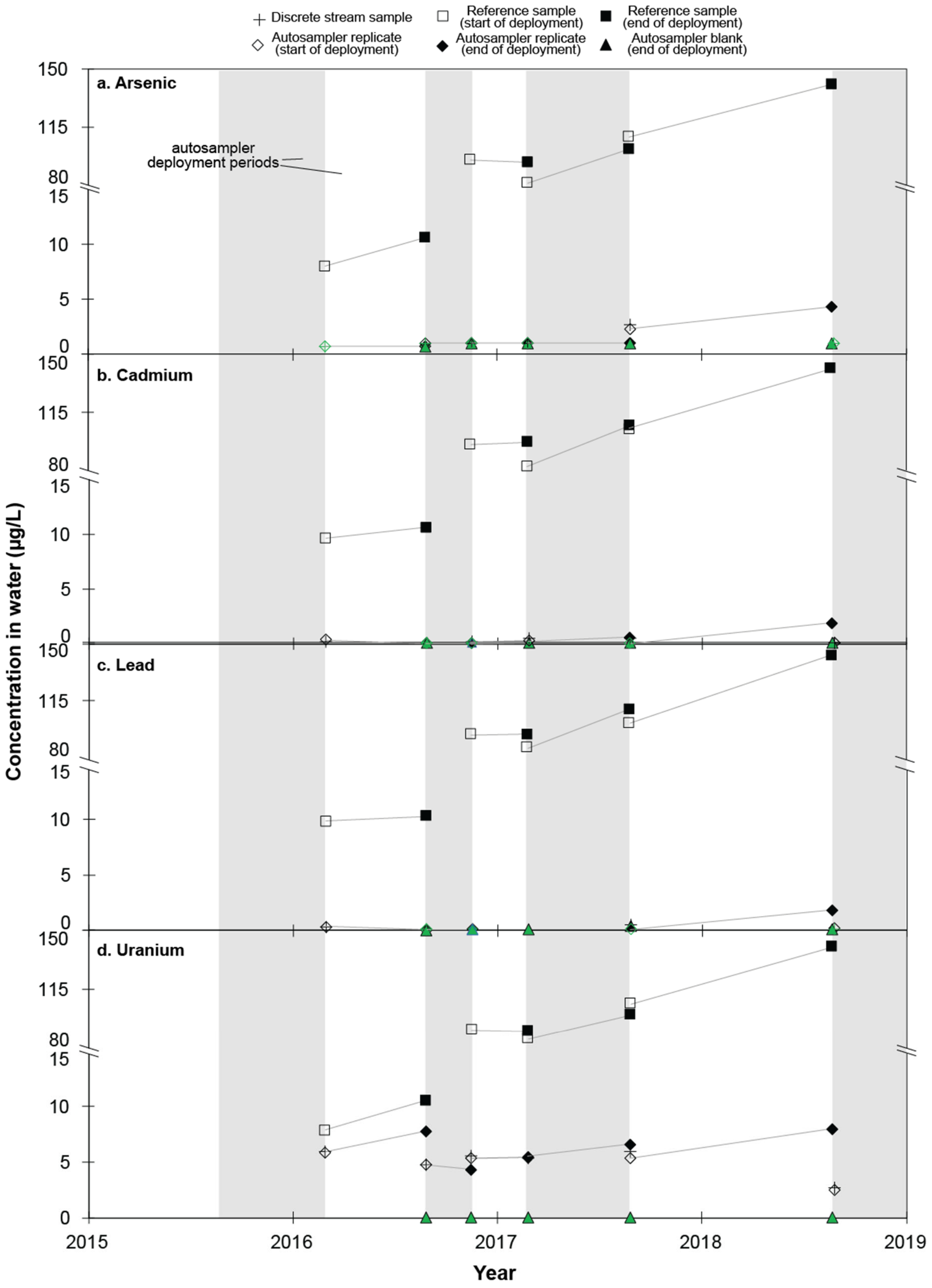

S12 Fig. Results from quality control (QC) samples at the Kanab Creek monitoring site. Symbols in green indicate values below reporting limits and are plotted at one-half the reporting limit. Lines drawn to connect pre- and post-deployment QC sample pairs do not imply a linear concentration change over the deployment.

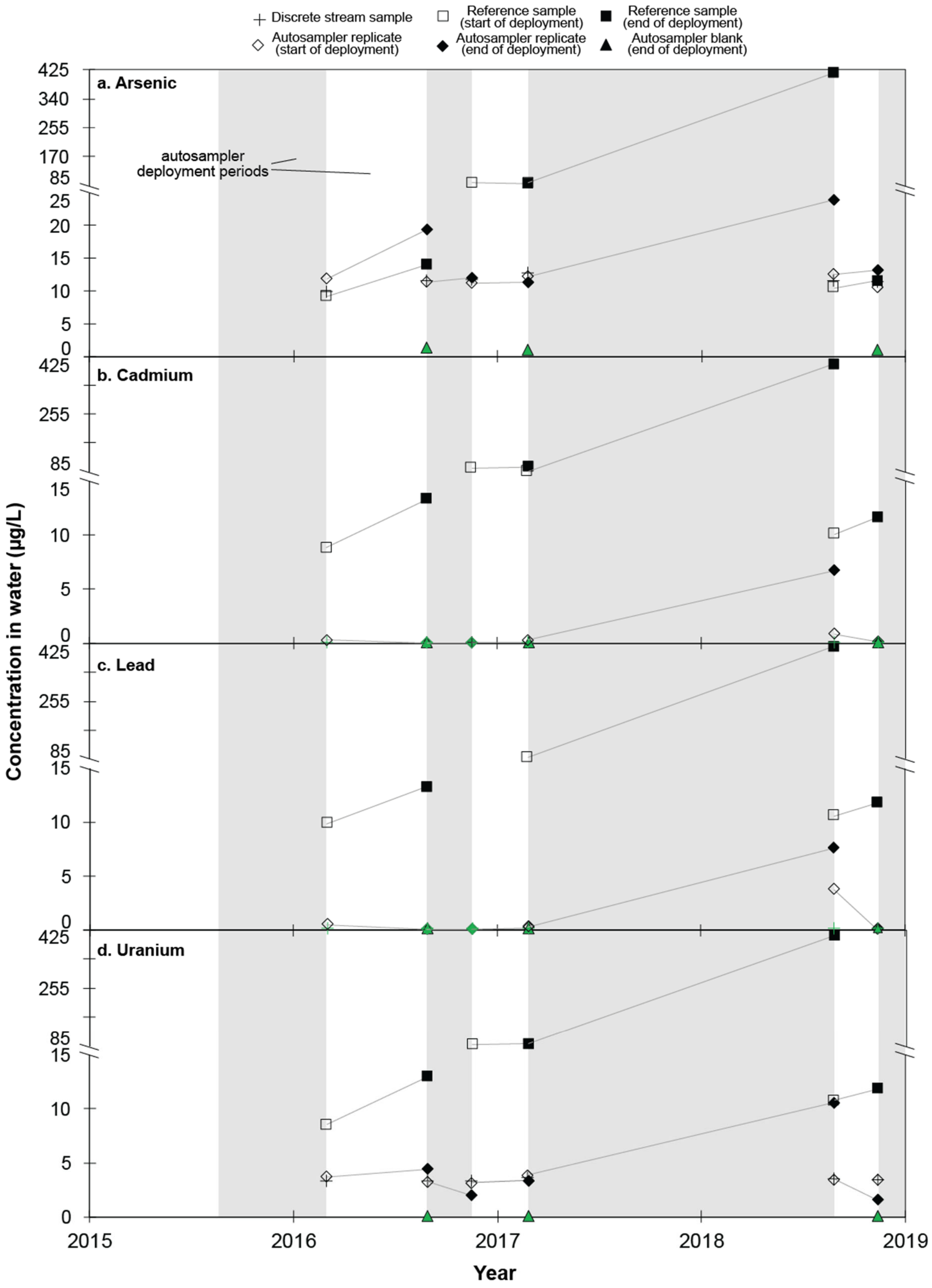

S13 Fig. Results from quality control (QC) samples at the Havasu Creek monitoring site. Symbols in green indicate values below reporting limits and are plotted at one-half the reporting limit. Lines drawn to connect pre- and post-deployment QC sample pairs do not imply a linear concentration change over the deployment.

S1 Text. Assessing the potential partitioning of uranium and select trace elements associated with breccia pipe uranium deposits between suspended sediment and water in autosampler bottles

Autosamplers were used to sample tributary sites because the remoteness of the locations permits only limited access (normally twice per year) by boat trips on the Colorado River. The limited access to sampling sites leads to potentially long time periods between sample collection and retrieval. Mixtures of suspended sediment and water inside sample bottles may potentially undergo partitioning that result in their concentrations being different at time of sample processing than what would be observed at time of sample collection. To assess the potential for partitioning between suspended sediment and water in autosampler bottles, two experiments were conducted in the lab.

Sediment-to-Water Experiment Methodology

Two suspended sediment samples from the Little Colorado River above the mouth near Desert View monitoring site were used for this experiment, one from 7/30/2017 and one from 7/22/2018. These samples were chosen because of their relatively large mass of sediment compared with other samples. First, the suspended sediment samples were air-dried. The dried sediment was crushed with a clean, acid-rinsed mortar and pestle and divided into two roughly equal portions. One portion was reserved as a control. The remaining portion of sediment was added to a cleaned and acid-rinsed 1L ISCO autosampler sample bottle and filled with 800 mL of deionized (DI) water. A filtered water sample was collected by syringe from the DI water before being added to the sediment. The autosampler bottle was capped and shaken vigorously, then allowed to sit for 3-4 weeks. At the end of the contact time, a filtered water sample was collected by syringe from the autosampler bottle. The bottle was then decanted of most of the liquid, with the remaining sediment slurry poured into pre-cleaned 600 mL polypropylene beakers and allowed to air dry. Pre- and post-experiment water samples were shipped to the USGS Geology, Geophysics, and Geochemistry Science Center Laboratory in Denver, Colorado for analyses. The dried sediment from the autosampler bottle and the reserved control sample were sent to the USGS contract laboratory AGAT Laboratories for elemental analysis.

Water-and-Sediment-Exchange Experiment Methodology

To evaluate the potential for partitioning between sediment and water in autosampler bottles, time series samples were collected from a single autosampler bottle. An autosampler bottle was filled by hand (dunking) at the Little Colorado River above the mouth near Desert View monitoring site on April 26, 2019 and a filtered water sample was collected by syringe immediately from the bottle. The bottle was then capped and transported back to the laboratory, where additional filtered samples were collected by syringe about every 2 weeks. After two months, all filtered water samples were shipped to the USGS Geology, Geophysics, and Geochemistry Science Center Laboratory in Denver, CO for analyses.

Results

Results for both de-ionized water – suspended sediment contact experiments indicate no measurable concentrations of target elements in de-ionized water prior to mixing with suspended sediment and unmeasurable or small amounts of target elements in water after contact with the sediment (S5 and S6 Tables). Arsenic contributed the greatest amount of mass to the aqueous phase in both experiments, with 1.9 and 2.7 µg of arsenic entering solution.

Time series samples from the natural-water filled autosampler bottle indicated little to no change in water phase concentrations of cadmium, lead, or uranium during the 2 months of the experiment (S7 Table). Concentrations of arsenic in water declined by almost 13% by the end of the experiment, but changes in arsenic were irregular during the experiment, with concentrations declining by 23% in the first month before recovering somewhat (S7 Table).

S5 Table. **Results of experiment to estimate partitioning from suspended sediment to de-ionized water.** Aqueous concentrations of less than reporting limits calculated as zeros.

	Arsenic		Cadmium		Lead		Uranium	
	W[1]	S[1]	W	S	W	S	W	S
Initial mass (µg)	<1	58.9	<0.02	0.8	<0.05	134.4	<0.1	18.2
Final mass (µg)	1.9	58.2	<0.02	0.9	0.2	133.1	0.2	17.7
% recovery	102%		117%		99%		98%	

[1]W is water (aqueous) phase, S is sediment (solid) phase.

S6 Table. **Results of repeat experiment to estimate partitioning from suspended sediment to de-ionized water.** Aqueous concentrations of less than reporting limits calculated as zeros.

	Arsenic		Cadmium		Lead		Uranium	
	W[1]	S[1]	W	S	W	S	W	S
Initial mass (µg)	<1	86.2	<0.02	1.9	<0.05	212.8	<0.1	41.8
Final mass (µg)	2.7	82.3	<0.02	2.0	<0.05	213.9	0.39	44.2
% recovery	99%		106%		101%		107%	

[1]W is water (aqueous) phase, S is sediment (solid) phase.

S7 Table. **Results of time-series experiment with natural water and suspended sediment.**

Date of sample collection	Arsenic µg/L	Cadmium µg/L	Lead µg/L	Uranium µg/L
4/26/2019	7.2	<0.2	<2	3.3
5/9/2019	7.3	<0.2	<2	3.3
5/28/2019	5.5	<0.1	<0.25	3.1
6/17/2019	6.2	<0.1	<0.25	3.2
6/28/2019	6.3	<0.1	<0.25	3.2

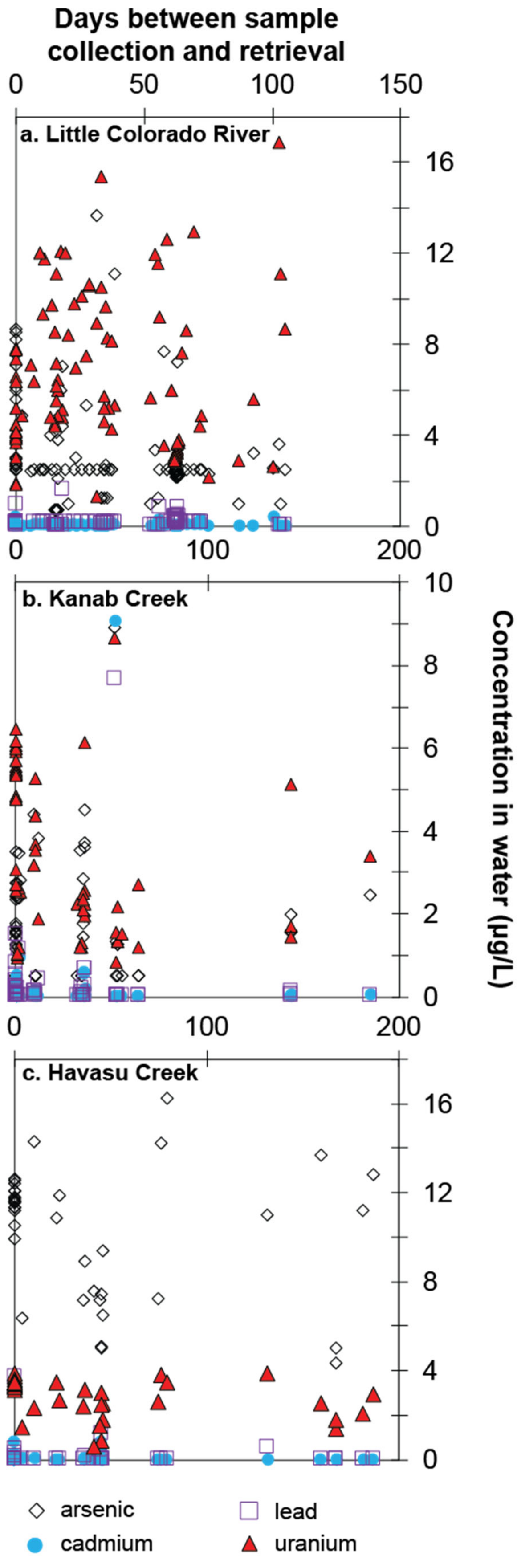

S14 Fig. Tributary sample concentrations plotted against the amount of time between sample collection and sample retrieval.

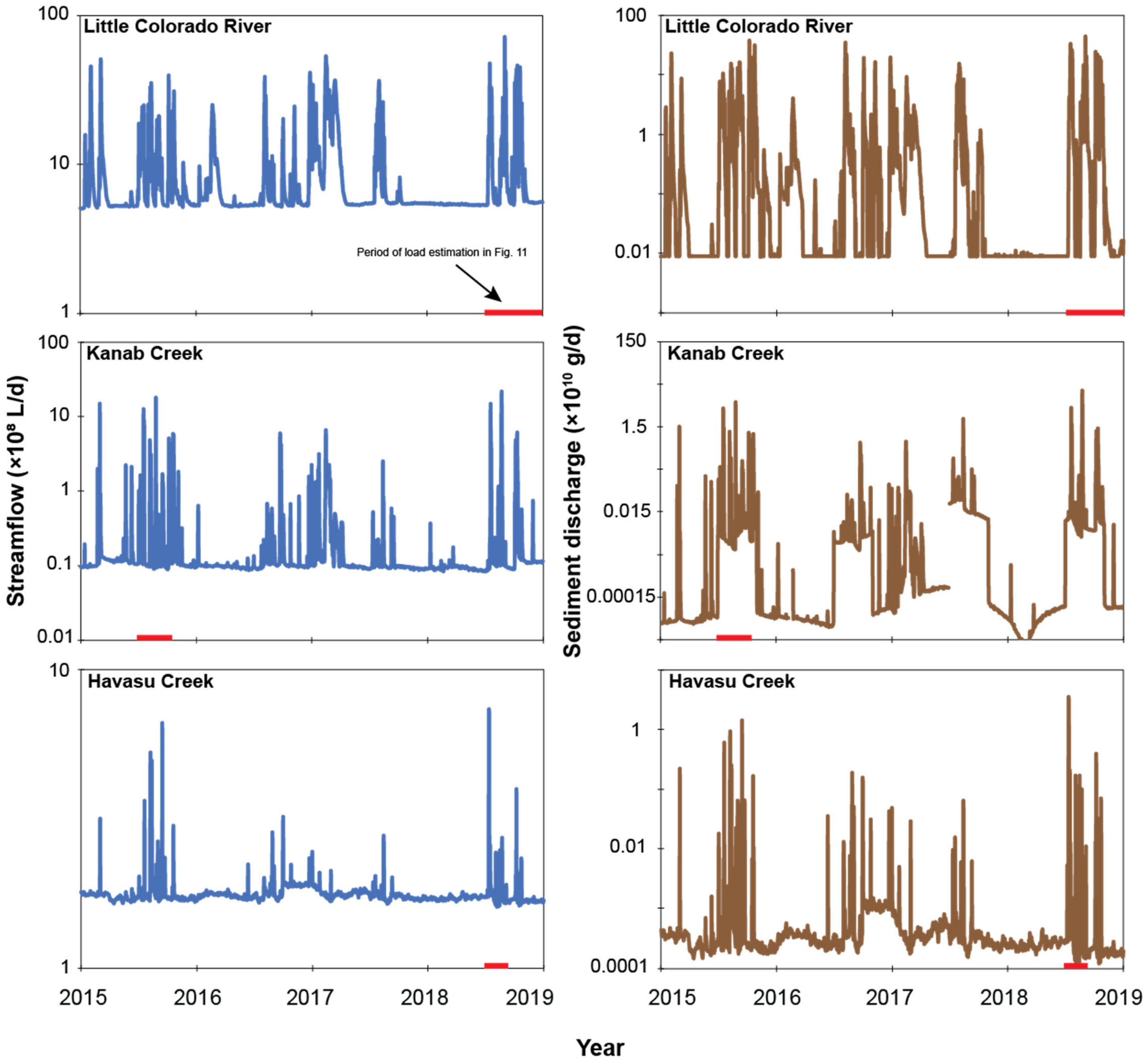

S15 Fig. Streamflow [26] and sediment discharge [30] data for the Little Colorado River, Kanab Creek, and Havasu Creek monitoring sites during the 2015–2018 study period.
